# Preclinical development of engineered biomaterial-based artificial bladder demonstrating core functions in a large-animal orthotopic model

**DOI:** 10.1016/j.mtbio.2026.103404

**Published:** 2026-06-29

**Authors:** Gyujun Choi, Jinho Kim, Won-Gun Koh, Jaehoon Jung, Ryounghoon Jeon, Jung Bae Seong, Youngjeon Lee, Taehyeon Kim, Wonkeun Park, Jeonghyeop Son, Bowoong Heo, Soojin Park, Jongwon Kim, Jeong-Mu Cheon, JunJie Piao, Yun-Hee Lee, Jongbaeg Kim, U-Syn Ha

**Affiliations:** a School of Mechanical Engineering, Yonsei University, 50 Yonsei-ro, Seodaemun-gu, Seoul, 03722, Republic of Korea; b Department of Urology, Seoul St. Mary's Hospital, College of Medicine, The Catholic University of Korea, 222 Banpo-daero, Seocho-gu, Seoul, 06591, Republic of Korea; c School of Mechanical Engineering, Yeungnam University, 280 Daehak-ro, Gyeongsan, 38541, Republic of Korea; d Department of Chemical and Biomolecular Engineering, Yonsei University, 50 Yonsei-ro, Seodaemun-gu, Seoul, 03722, Republic of Korea; e Daegu-Gyeongbuk Medical Innovation Foundation, 88 Dongnae-ro, Dong-gu, Daegu, 41061, Republic of Korea; f National Primate Research Center, Korea Research Institute of Bioscience and Biotechnology, 30 Yeongudanji-ro, Ochang-eup, Cheongwon-gu, Cheongju-si, Chungcheongbuk-do, 28116, Republic of Korea

**Keywords:** Artificial bladder, Engineered biomaterial, Restoration of bladder function, Urine storage, Urine voiding

## Abstract

This study demonstrates an artificial bladder that restores urine storage, sensing, and controlled voiding by integrating engineered biomaterials. It comprises a main body for urine storage, a pressure control system for voiding, and a wireless, battery-free urine fullness sensor. The main body consists of an outer wall and a thin septum membrane. The outer wall is fabricated using poly(dimethylsiloxane)(PDMS) and Ecoflex (Smooth-On, Inc.) to achieve both mechanical strength and flexibility, with a Parylene-C coating ensuring biocompatibility. Inside, a diagonally positioned septum membrane separates the urine storage and working fluid compartments. Reinforced with a glass fiber mesh, this membrane allows controlled deformation and stable pressure regulation. The sensor, integrated into the bladder wall, enables wireless urine volume monitoring without an internal power source. The outer wall and pressure control system, including a manual pump and on/off valve, are fabricated using 3D-printed molds. In vitro simulation and in vivo experiments with mini pigs for 6 weeks demonstrated that the artificial bladder mimics human bladder function successfully, while maintaining stable storage pressure and generating sufficient voiding pressure via an external pump. Post-implantation analysis confirmed biocompatibility, structural stability, and secure urinary tract connection. These findings highlight the potential of biomaterial-based artificial organs in restoring bladder function.

## Highlights

• Engineered biomaterial-based artificial bladder restores storage and voiding.
• Glass fiber–reinforced septum enables low-pressure urine storage.
• Battery-free RFID sensor wirelessly detects bladder fullness.
• Stable urodynamic performance demonstrated in a pig orthotopic model.
• Six-week in vivo study confirms biocompatibility and structural integrity.

## Graphical abstract

**Figure:**
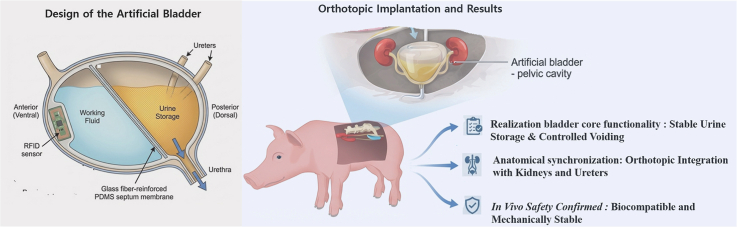

## 1 Introduction

The bladder, a vital organ in the human body, has a relatively simple structure and function compared to other organs involved in complex biochemical processes. Its primary role is to store and expel urine, which is continuously produced by the kidneys. Despite its seemingly straightforward function, proper bladder operation is essential for maintaining overall health and quality of life. Bladder dysfunction, whether caused by bladder cancer requiring cystectomy or neurological damage, can significantly reduce quality of life and necessitate major lifestyle adjustments [[1], [2], [3], [4]].

These challenges highlight the urgent need for effective rehabilitation strategies to restore bladder function [5,6]. Despite advancements in medical science, current treatment options remain limited. Traditional approaches have focused on tissue replacement or partial functional supplementation but have not yet succeeded in fully restoring bladder function [[7], [8], [9], [10], [11], [12]].

From a clinical perspective, intestinal segments have been used as passive urine reservoirs in bladder reconstruction. However, they lack the ability to sense bladder fullness and cannot actively generate the pressure needed for controlled urine discharge, a key function of the native bladder [[13], [14], [15], [16], [17]]. Furthermore, intestinal segments are prone to complications, including infections and metabolic disturbances, making them suboptimal for long-term bladder replacement [13,[18], [19], [20]]. In regenerative medicine, tissue engineering efforts have attempted to develop bladder tissues using biomaterials and cell-seeding techniques [8,10,[21], [22], [23], [24], [25], [26], [27], [28], [29]]. However, these approaches face challenges such as poor tissue integration, insufficient mechanical strength, and an inability to fully replicate the bladder's dynamic environment. Additionally, research has explored methods to restore specific bladder functions, such as urine fullness sensing [[30], [31], [32], [33]]. Technologies utilizing pressure sensors or electrical impedance have been developed to monitor bladder volume and provide alerts for voiding [31,[34], [35], [36], [37]]. While these innovations represent progress, they fail to fully replicate the bladder's complex interaction between storage and controlled voiding mechanisms.

Given the importance of bladder function and the limitations of current treatment options, there is an urgent need for innovative solutions that restore full bladder functionality. To address this, we developed an artificial bladder that integrates engineered biomaterials. This system is designed to replicate key physiological functions - urine storage, sensing, and controlled voiding - while overcoming the structural and functional limitations of existing approaches. To achieve this, the main body was fabricated using a composite of PDMS and Ecoflex, providing both mechanical strength and flexibility, with a Parylene-C coating ensuring biocompatibility. A glass fiber-reinforced septum membrane was incorporated to regulate pressure changes, mimicking the bladder's natural response to filling and voiding. Additionally, 3D-printed molds were used for precise fabrication of the outer wall and voiding pressure control components, ensuring structural integrity and functional reliability. The system also includes a wireless, battery-free radio-frequency identification (RFID) sensor for real-time urine volume monitoring. By integrating biomaterial engineering, 3D printing-assisted fabrication, and smart sensing technology, this study presents a novel approach to artificial bladder development. Both in vitro and in vivo results demonstrate the system's potential as a functional and biocompatible solution for bladder dysfunction, offering new possibilities for clinical translation in the field of organ prosthetics.

## 2 Materials and methods

### 2.1 Fabrication of the main body of the artificial bladder

The main body of the artificial bladder consists of an outer wall and a thin septum membrane. The outer wall is divided into two sections, with the septum membrane positioned diagonally from the dorsal to the ventral side, creating two distinct compartments: the working fluid and urine storage spaces.

The outer wall was fabricated using a molding process, starting with a master mold 3D-printed from acrylonitrile butadiene styrene (ABS), which was then used to create a silicone (Smooth Sil-950, Smooth-on) replica mold. The working fluid compartment included two 6 mm diameter holes for fluid connection, while the urine storage compartment featured a 10 mm diameter hole for urethral connection and two 5 mm diameter holes for ureteral connection. The outer wall was made from PDMS with a 5:1 base-to-curing agent ratio, blended with Ecoflex 00-30 at a 10:1 wt ratio to enhance flexibility.

The septum membrane was fabricated by spin-coating PDMS onto a glass fiber mesh fixed on a 21 cm diameter disk to ensure uniform coverage. The coating process was performed at 1000 rpm for 30 s. The PDMS-glass fiber mesh membrane was then floated on a water tank containing a submerged water transfer printing mold, and the water was drained to transfer the 2D pattern onto a curved 3D structure, enabling large-area membrane fabrication. The membrane was cured at 80 °C for 2 h, resulting in an 83 μm-thick flexible and durable septum membrane suitable for bladder applications.

### 2.2 Construction of the voiding pressure control system

Voiding pressure control system consists of manual pump for urination, on/off valve for controlling the flow of the working fluid and working fluid reservoir.

#### 2.2.1 Manual pump

Fig. S1 illustrates the manual pump, which consists of a pump body (upper part) and a base panel (lower part). The pump features two fluid inlets and a pressurization chamber. The pump body was molded using a 3D-printed poly(lactic acid)(PLA) die and cast with a 50:50 silicone (Dragon Skin-30) and hardener mixture, then cured at 80 °C for 4 h. With Shore A 30 hardness, it enables effective contraction and relaxation. The pump includes two 6 mm × 40 mm openings with silicone check valves for unidirectional flow. The flat base panel, made from the same silicone mixture, ensures efficient pressure transfer when implanted. Both parts were bonded with biocompatible Ecoflex™ 00-35 FAST adhesive for structural integrity.

#### 2.2.2 On/off valve

The on-off valve allows controlled flow only when operated by the user, unlike the check valve, which provides continuous flow. It consists of a valve pressure part, base panel, and internal connector, all molded from silicone (MED-4940) using a P20-Die Steel mold. The valve pressure part has 7.2 mm diameter fluid passages and a flexible top for controlled pressurization. The flat base panel ensures stability, while the internal connector features a 10 mm shield that blocks flow when pressed. Assembly involved molding the valve pressure part, inserting the connector, and securing it to the base panel with biocompatible Ecoflex™ 00-35 FAST adhesive. Pressing the valve during bladder drainage blocks fluid return (“off” state), and releasing it reopens the conduit (“on” state), reducing pressure and allowing fluid to return to the reservoir.

#### *2.2.3* Working fluid reservoir

The working fluid reservoir stores fluid to facilitate urination by transferring pressure to the artificial bladder. Made from DEHP-free medical-grade PVC with a Parylene-C coating for biocompatibility, it is designed to match the bladder's capacity. The edges are heat-sealed, with a fluid entry tube at the top, and any gaps sealed using a 50:50 mix of Ecoflex™ 00-35 FAST adhesive. Its two-way structure, with all fluid entry and exit points at the top, optimizes space within the body.

### 2.3 Urine fullness sensor

RFID technology, a wireless communication method, was used to monitor urine fullness without requiring an internal power source. The sensor, integrated into the artificial bladder's outer wall, consists of a variable capacitor, an RFID chip, and a coil antenna. The capacitor, initially designed with a curved electrode and fixed electrodes (Fig. S2a), changes capacitance as urine volume increases, altering the resonance frequency of the sensor circuit. This change is detected by an external RFID reader, enabling urine fullness measurement. Nickel fabric electrodes, chosen for their conductivity and flexibility, were coated with a thin PDMS layer to maintain a small gap when in contact. The capacitor electrodes, along with the RFID chip and antenna, were integrated into the outer wall mold, and the sensor assembly was completed by molding and wiring the curved electrodes (Fig. S2b).

### 2.4 Cytotoxicity assay

Cytotoxicity tests were performed on mouse fibroblasts to investigate the cytotoxicity of the artificial bladder. The test samples and extraction medium (1× MEM with 5% fetal bovine serum) were placed in a glass vessel and sealed. The test sample was extracted at 37 ± 1 °C for 70 rpm at 72 ± 2 h and then subjected to the test. The test sample extract was treated with each of three L-929 fibroblast culture wells that formed a monolayer and was incubated for 48 h at 37 ± 1 °C and 5 ± 1% CO2 in air. After 48 h of incubation, the ratio of round cells, loss of intracytoplasmic granules, lysed cells, and growth inhibition were observed under a microscope. If the negative and positive control sample groups met the criteria, and the grade of the test sample group was no more than 2, and the test sample was determined to be non-cytotoxic.

#### 2.4.1 Stability evaluation of the artificial bladder via accelerated aging test under simulated implantation conditions

The stability of the artificial bladder was evaluated through an accelerated aging test simulating one year of implantation under harsh conditions. To replicate real-time aging within a shortened timeframe, the stability of the artificial bladder was evaluated through an accelerated aging test conducted at 80 °C and 100% relative humidity. These conditions were selected to simulate a physiologically relevant moist environment under harsh conditions, while also preventing thermal deformation of the artificial bladder and ensuring effective acceleration of the aging process. Based on a real-time aging period of one year, the accelerated aging duration was calculated and set to 19 days. The stability of the artificial bladder was evaluated based on its operational reliability, assessed through the comparison of water input and output volumes. Normal function of the artificial bladder was confirmed when the input and output volumes deviated by no more than 5% from the input volumes.

### 2.5 In vitro urodynamic studies and sensing experiments

In vitro urodynamic studies were conducted to evaluate urine storage and voiding functions of the artificial bladder. A urodynamic testing device (Aquarius TT; Laborie Medical Technologies, Mississauga, ON, Canada), commonly used for human subjects, was employed in conjunction with the artificial bladder and voiding pressure control system. A pressure flow study was performed to measure bladder capacity, compliance, and internal pressure, key indicators of urine storage and voiding function. Yellow liquid was used as virtual urine, while blue liquid represented the working fluid to visualize the bladder's operating mechanism.

For in vitro sensing experiments simulating pig trials, pork belly tissue was used to mimic abdominal tissue as a medium between the sensor and RFID reader. Deionized water was used as the working fluid, with capacitance adjusted accordingly. The sensor was designed to activate when urine volume reached a set threshold, triggering an alert for bladder emptying. Notifications were configured to be delivered through vibration, sound, or light via a patch-type reader attached to the abdomen or a mobile phone application via Bluetooth. To validate the system's performance, experiments assessed wireless communication range through biological tissue and measurement accuracy of urine volume detection. These tests ensured reliable signal transmission and precise urine volume detection, confirming the system's functionality for practical application.

### 2.6 In vivo implantation and assessment

This animal study was approved by the Daegu Gyeongbuk Medical Innovation Foundation Care and Use Committee (KMEDI-24031202-01). The animals received humane care in accordance with the Guide for the Care and Use of Laboratory Animals (Institute of Laboratory Animal Resources, Commission on Life Sciences, National Research Council, National Academy Press, Washington, DC, USA, 2011) and institutional guidelines.

An artificial bladder was implanted in a pig under general anesthesia using the standard transabdominal method. A midline incision was made to expose the pelvic area and ensure a clear surgical view. The bilateral ureters and urethra were carefully dissected and secured before detaching the native bladder (Fig. S3a). The artificial bladder was then placed in the same orthotopic position as the native bladder on the pelvic floor (Fig. S3b). The ureters were sutured to the inlet annulus of the artificial bladder, while the urethra was connected to the outlet annulus, with a Foley catheter inserted and kept in place until the end of the experiment (Fig. S3c). After controlling bleeding and placing a drain tube on the pelvic floor, the abdominal incision was closed (Fig. S3d). Postoperative care, including hydration, infection prevention, nutritional support, wound care, and monitoring, was provided according to standard protocols until the conclusion of the experiment. A computed tomography (CT) scan was performed two weeks post-implantation to assess the general condition of the abdominal cavity and verify the stable connection of the upper and lower urinary tracts. To evaluate post-implantation safety, parameters such as weight and blood tests (renal function, liver function, and electrolyte levels) were monitored weekly and compared with pre-implantation values.

Six weeks post-implantation, a urodynamic evaluation of the artificial bladder was conducted under general anesthesia. The urodynamic evaluation consisted of two phases: urine storage and voiding. A cystometrogram was used to record the intravesical pressure within the urine storage partition using a polygraph system (Grass 7D, Grass Institute Co., Quincy, MA, USA), while saline was infused at a rate of 10 mL/min via a syringe pump (Harvard Apparatus, Holliston, MA, USA). Because coordinated voluntary relaxation of the urethral sphincter cannot be realistically reproduced in pigs, the urodynamic evaluation was performed under controlled drainage conditions. A Foley catheter placed within the artificial bladder was connected to a threshold valve set at 50 cmH_2_O, allowing pressure-driven urine drainage once the predetermined filling pressure was reached. Intravesical pressure was continuously recorded during filling and voiding, and both infused and voided volumes were measured to calculate voiding efficiency.

Following completion of all experiments, the artificial bladder was explanted from each pig to assess structural stability. Parameters from the urodynamic evaluation were measured and analyzed. Except for functional bladder capacity, which was experimentally determined, target values for the remaining parameters were selected to approximate reported physiological ranges observed in healthy adult humans [38,39]. Voiding efficiency was defined as the ratio of the effective voiding volume to the functional bladder capacity, with functional bladder capacity representing the storage volume corresponding to the point of normal urinary desire.

### 2.7 Evaluation of calcification on explanted artificial bladder surfaces using Alizarin Red S staining

After 6 weeks of implantation, the artificial bladder was explanted and the urine and working fluid compartments were separated. Internal surfaces were gently rinsed with deionized water, and representative sections were dried at 80 °C. Samples were immersed in 20 mg/mL Alizarin Red S (Sigma–Aldrich) for 45 min at room temperature, then treated with cetylpyridinium chloride to extract the bound dye. Absorbance was measured at 550 nm using a microplate reader (VERSA Max, Molecular Devices).

### 2.8 Statistical analysis

Data for the urinary calcification analysis are presented as mean ± standard deviation (n = 5). Statistical comparisons between groups were performed using an unpaired two-tailed Student's t-test, and differences were considered statistically significant at p < 0.05.

## 3 Results

### 3.1 Design and working principle of artificial bladder

**Fig. 1:**
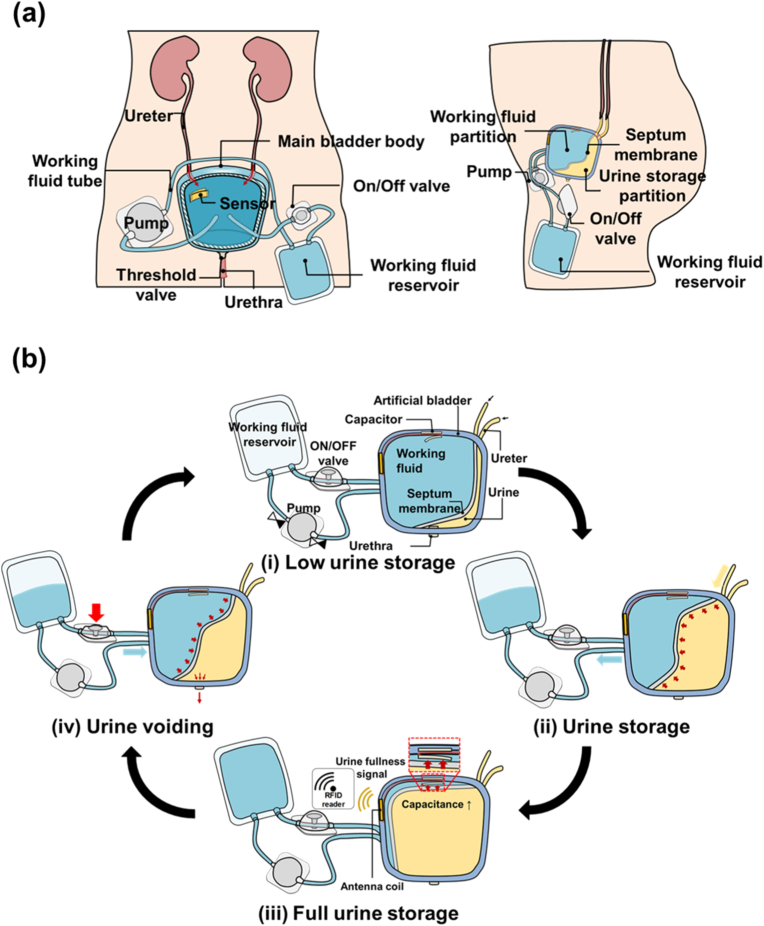
**Design, components and working principle of the proposed artificial bladder.** (a) Schematic of the artificial bladder system, consisting of three main components: the main bladder body, voiding pressure control system, and urine volume sensing system. (b) Illustration of the storage and voiding functions. As urine fills the storage partition, the septum membrane is pushed, which displaces the working fluid into the reservoir. Voiding occurs when the manual pump forces the working fluid back into the working fluid partition, which moves the septum membrane dorsally and creates pressure for urine discharge. A check valve ensures unidirectional flow. Here, the battery-less, RFID-based sensor detects when the urine volume exceeds a set threshold, alters the capacitance in the embedded capacitor, and triggers a wireless signal to an external reader.

The proposed artificial bladder system is composed of three components: the main bladder body, voiding pressure control system, and urine volume-sensing system (Fig. 1a). First, the main body was designed with two partitioned spaces separated by a diagonally positioned septum membrane, extending from the dorsal to ventral side of the bladder. The dorsal partition serves as the urine storage area, while the ventral partition accommodates the working fluid, which generates voiding pressure. The urine storage partition is connected to the ureter and urethra via commercially available vascular grafts, whereas the working fluid partition is linked to the voiding pressure control system through a silicone tube. Second, the voiding pressure control system includes a manual pump for voiding, an on/off valve, and a working fluid reservoir. Third, the urine volume-sensing system utilizes battery-free wireless communication based on RFID technology, which alerts the user when the artificial bladder reaches a certain urine volume, indicating the need to void.

The urinary bladder performs three main physiological functions: urine storage, fullness sensing, and voiding. The artificial bladder was designed to mimic these key functions and closely replicate human physiology (Fig. 1b). As urine enters the urine storage partition, the septum membrane is pushed ventrally due to pressure from urine accumulation, creating storage space. This movement generates driving pressure, forcing the working fluid into the reservoir. The septum membrane is designed to exhibit low elastic recovery force while enabling effective deformation under low pressure. To verify the design and deformation behavior of the septum membrane, hydrostatic loading tests were performed by filling the urine storage chamber under controlled pressure conditions. As shown in Movie S1, the septum membrane deformed with minimal resistance in response to fluid accumulation. Notably, urine storage was achieved with only a few centimeters of H_2_O pressure, demonstrating the membrane's high compliance and low-threshold deformation characteristics. This result confirms that the septum membrane readily accommodates incoming urine under physiologically relevant pressure conditions.

Supplementary data related to this article can be found online at https://doi.org/10.1016/j.mtbio.2026.103404

The following are the Supplementary data related to this article:

Multimedia component 3

When the urine storage partition reaches its threshold volume, the movement of the septum membrane activates an embedded capacitor located in the ventral wall of the main body. This change triggers an external alarm module, mimicking the bladder's natural fullness sensing mechanism.

Effective voiding requires generating pressure within the bladder. Pressing the manual pump transfers the working fluid back into the ventral partition, displacing the septum membrane dorsally and generating sufficient pressure for urine expulsion. To ensure one-way flow and prevent backflow, a one-way check valve is integrated into both the on/off valve and the pump. The pump itself is designed with a threshold pressure, allowing fluid movement only during intentional activation. The user manually operates the pressure for urine discharge, while urine fullness detection is performed through wireless communication, eliminating the need for an internal battery. This design allows the artificial bladder to function long-term without requiring battery replacement. Movie S2 illustrates the working principle of this artificial bladder.

Supplementary data related to this article can be found online at https://doi.org/10.1016/j.mtbio.2026.103404

The following are the Supplementary data related to this article:

Multimedia component 4

### 3.2 Fabrication of the main body of the artificial bladder

The main body of the artificial bladder was fabricated by bonding two outer wall structures—one forming the urine storage partition and the other forming the working fluid partition—together with a centrally positioned septum membrane.

In healthy adults, urine is produced at a rate of approximately 30–50 mL per hour, resulting in a total daily output of 1200∼1500 mL, which is typically voided about eight times per day. The average volume per void at the point of normal urinary desire is reported to be approximately 150∼200 mL. In individuals with increased voiding frequency, the volume may be closer to 100 mL per episode.

Based on these physiological values, the in vitro artificial bladder model was designed with a target functional capacity of 200 mL, which falls within the clinically relevant range for a single voiding event. For the in vivo pig model, however, the reservoir volume was intentionally scaled down because the pelvic cavity of the experimental pig was considerably smaller than that of an adult human. Under these anatomical constraints, the implanted prototype achieved a functional capacity of approximately 100 mL and demonstrated an effective voiding volume of approximately 89 mL.

Therefore, the voiding volume observed in the pig model should be interpreted as the functional outcome of an anatomically scaled large-animal prototype, rather than as the final target capacity of a human artificial bladder. Within the spatial limitations of the pig pelvic cavity, the device demonstrated in vivo urine storage and controlled voiding, supporting the feasibility of further anatomical scaling and optimization for human application.

For future human use, the system is expected to be configured with a larger functional reservoir volume, potentially exceeding 150 mL, based on adult pelvic anatomy and clinically relevant voiding volumes. Although the working fluid reservoir increases the total implant volume, the system is designed to distribute its components across separate anatomical compartments, with the artificial bladder located in the pelvic cavity and the working fluid reservoir placed in the subcutaneous abdominal space. This compartmentalized design may help reduce localized volume burden while preserving urine storage capacity and overall functional performance.

The artificial bladder implanted in the mini pig model had a maximum diameter of 80 mm, a height of 70 mm, and a functional capacity of approximately 100 mL. These dimensions were selected considering the anatomical constraints of the mini pig pelvic cavity. In addition, based on anatomical information obtained from abdominal imaging and surgical observations of the mini pig model, the artificial bladder was designed with a truncated-cone geometry rather than a simple spherical structure. This geometry was selected to better conform to the available pelvic and lower abdominal space while minimizing interference with adjacent organs and surrounding tissues. The tapered shape also facilitates anatomical placement within the limited implantation space and improves spatial compatibility between the bladder body and associated components of the artificial bladder system. These design considerations were incorporated to maximize functional bladder capacity while maintaining safe implantation within the anatomical constraints of the animal model.

**Fig. 2:**
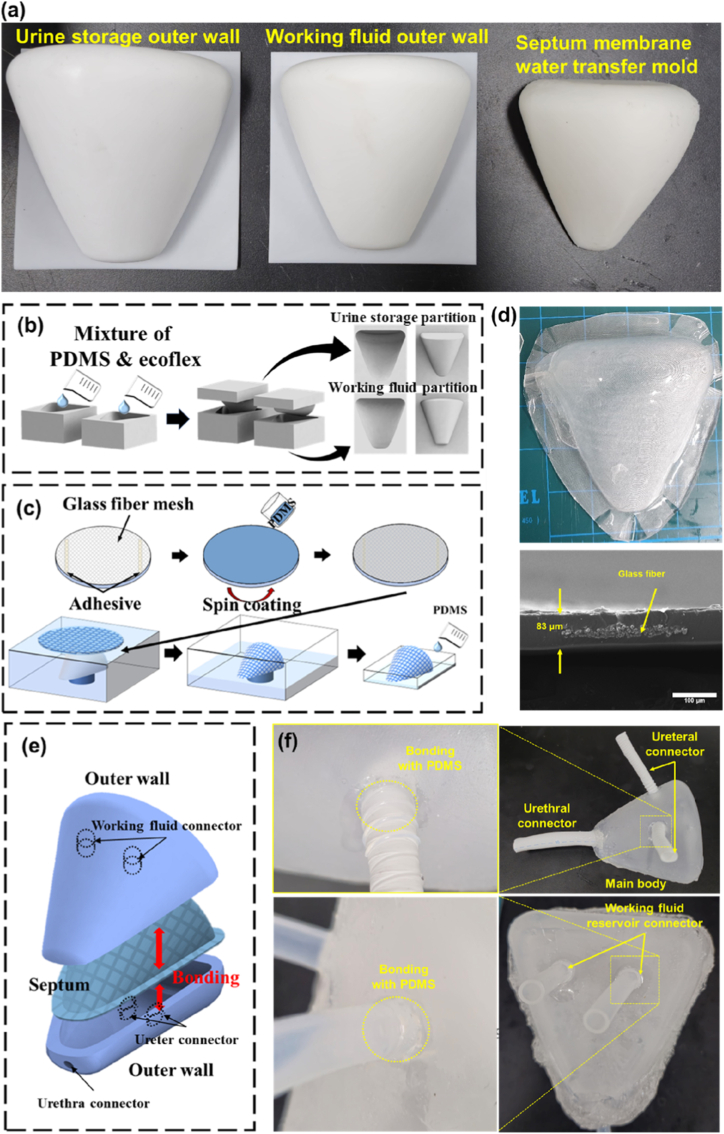
**Fabrication of main body of artificial bladder.** (a) Photographs of outer wall master mold and water transfer printing mold for the septum membrane, both produced via 3D printing. (b) Schematic illustrating the molding process for the outer wall of the artificial bladder using mixture of PDMS and Ecoflex. The mixture is poured into molds to form the bladder's outer walls, which are then cured to achieve the required shape and flexibility. (c) Process for fabricating the septum membrane, which includes reinforcing it with a glass fiber mesh and shaping it into a 3D structure using water transfer printing. (d) Images of the septum membrane: (top) photo of the fabricated septum membrane, and (bottom) a scanning electron microscope (SEM) image of the cross-section of glass fiber-embedded PDMS membrane, showing fiber integration within the 83 μm-thick membrane. (e) Schematic illustration of assembling the main body of artificial bladder, showing the outer walls, septum membrane, and connection points for working fluid, ureter, and urethra. (f) Photographs of the main body of the artificial bladder, showing the assembly of ureteral and urethral connectors, as well as the conduit connecting to the working fluid reservoir, all bonded to the outer wall of the urine storage partition using PDMS.

Since the size and shape of the artificial bladder vary for each patient, 3D printing was used to fabricate the master mold for the outer wall. The master mold shown in Fig. 2a was produced using fused deposition modeling (FDM)-based 3D printing and was then used to create a silicone replica mold for outer wall fabrication. Fig. 2b illustrates the molding process for the two outer walls of the artificial bladder using a PDMS and Ecoflex mixture. To ensure stability within the body, the artificial bladder must be securely anchored to the fat layer of the pubic bone, requiring an outer wall that is both rigid and durable to prevent deformation. To achieve this, the outer wall was made of PDMS with a higher curing agent ratio (5:1) to enhance rigidity. At the same time, the bladder must withstand high abdominal pressure without tearing, necessitating a certain degree of flexibility. To balance these properties, Ecoflex was blended with PDMS, improving elasticity while maintaining structural integrity [40]. This combination ensures that the artificial bladder remains mechanically stable under external forces while retaining the flexibility needed for long-term functionality.

The septum membrane must be highly deformable to accommodate changes in urine volume, minimizing pressure exerted on the kidneys while also preventing negative pressure generation inside the artificial bladder after urine expulsion by reducing elastic recovery force. As shown in Fig. 2c, the septum membrane was fabricated by coating a glass fiber mesh with PDMS. The mesh, with a thickness of 45 μm and a pitch of 50 μm, maintains high flexibility and tensile strength without excessive stretching. This design optimizes the flexibility of both PDMS and the glass fiber mesh, while the mesh structure effectively suppresses PDMS's elastic recovery force, preventing the formation of negative pressure inside the bladder. Additionally, the glass fiber mesh enhances the durability of the thin membrane. To achieve a 3D structure with a larger surface area, the water transfer printing method was used, preventing folds in the membrane while ensuring full deformation according to urine storage capacity. The final septum membrane, with a thickness of 83 μm, was internally embedded with glass fibers and produced in a 3D shape that could be easily modified to fit the outer wall of the artificial bladder (Fig. 2d). The incorporation of glass fibers within PDMS was confirmed by cross-sectional SEM imaging (Fig. 2d). Finally, as shown in Fig. 2e, the two outer walls and the septum membrane were bonded using PDMS, completing the fabrication of the artificial bladder's main body. Fig. 2f presents the assembled artificial bladder, where the urine storage partition includes three connectors: two ureteral connectors (5 mm in diameter each) and one urethral connector (10 mm in diameter). The spacing between the ureteral connectors was designed to be 45 mm to match the ureteral gap in experimental pigs. The ureteral connectors, made of GORE-TEX®, were bonded to pre-formed holes in the outer wall using PDMS. For the working fluid partition, two connectors were required: one conduit for fluid movement from the working fluid reservoir to the artificial bladder and one conduit for fluid return to the reservoir. Silicone rubber tubes were used for these connections.

All components of the artificial bladder, including the outer walls and septum membrane, were conformally coated with a 3 μm-thick Parylene-C layer prior to assembly. This coating enhances biocompatibility and provides additional mechanical stability to the structure by forming a uniform barrier over all internal and external surfaces of artificial bladder [[41], [42], [43], [44]].

### 3.3 Mechanical durability and interface stability

**Fig. 3:**
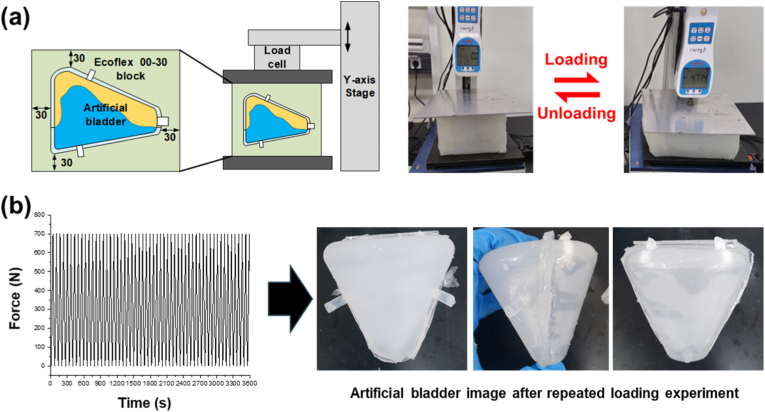
**Mechanical durability and sealing stability of artificial bladder.** (a) Schematic of the durability test set-up for the artificial bladder, designed to simulate substantial external pressure. The bladder is positioned within a support structure with 3 cm of Ecoflex 00-30 padding on all sides, which mimics the physical properties of pork belly to provide realistic fixation and support. The set-up includes connectors for working fluid, ureter, and urethra to reflect anatomical placement and functionality. To replicate conditions where the bladder might endure significant loads, a load cell applies vertical force on the bladder assembly. This configuration aims to evaluate the bladder's structural integrity and resistance to external forces, ensuring robustness against potential damage scenarios such as abdominal compression, which could otherwise result in cracking or separation at bonded areas. (b) Repetitive mechanical loading tests simulating the body weight of a mini pig (equivalent to in vivo conditions) were conducted over 1000 cycles. Real-time load monitoring ensured the immediate detection of structural defects. Results demonstrated no leakage, delamination, or interface failure, confirming the artificial bladder's mechanical robustness and long-term sealing stability under cyclic compressive stress. This validates its reliability for sustained biological applications.

Ensuring sufficient durability is crucial for the effective in vivo application of an artificial bladder. If the artificial bladder lacks durability, external forces can cause the outer wall to crack or the bonded areas to separate, which could lead to the leakage of stored urine and working fluid into the surrounding tissues. To mitigate this risk, durability tests were conducted prior to the animal studies to evaluate the resistance of the bladder to external forces. The greatest risk of damage occurs when a user lies on the ground or applies pressure to the abdomen, which subjects the bladder to a substantial load. A durability test was designed to simulate these conditions, as illustrated in Fig. 3a. Given the curved 3D shape of the artificial bladder, Ecoflex 00-30, which is a material with physical properties similar to those of pork belly, was used to secure the bladder. The artificial bladder was embedded within an Ecoflex 00-30 block, and force was applied to replicate the real conditions of the implanted bladder. Loads equivalent to the body weight of the mini pig used in the in vivo study were applied over 1000 cycles (Fig. 3b). During the test, the applied load was continuously monitored in real time, enabling immediate detection of any structural damage or delamination. The results confirmed that the bonding interfaces of the bladder and the bladder body itself remained structurally intact without leakage or failure throughout the test. These findings validate the mechanical robustness and interface stability of the artificial bladder under repeated compressive stress, supporting its reliability for long-term in vivo applications.

### 3.4 Voiding pressure control system operation

**Fig. 4:**
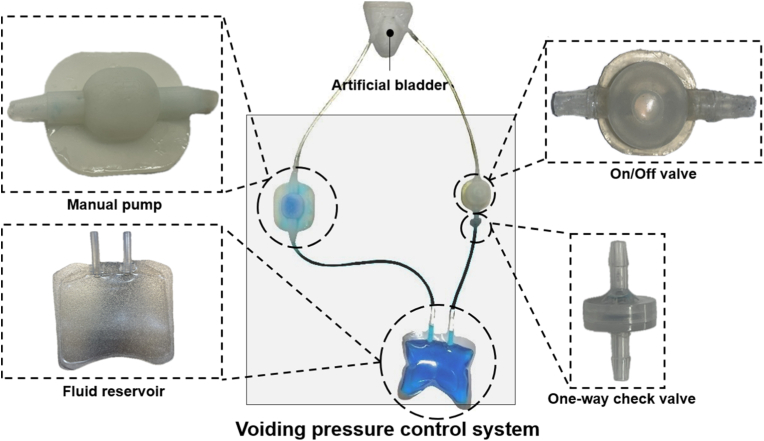
**Photograph and schematic of the voiding pressure control system connected to the artificial bladder.** The system included a manual water pump to transport the working fluid, an on/off valve to control the fluid flow, a check valve to ensure unidirectional flow, and a fluid reservoir storing the working fluid. The pump generates the internal pressure required for voiding by transferring fluid into the working fluid reservoir.

The manual pump, on/off valve, and fluid reservoir were successfully fabricated and integrated with the main body of the artificial bladder, completing the voiding pressure control system, as shown in Fig. 4. In the final system, the working fluid moved as intended, with precise control and no leakage.

The manual pump, essential for voiding, facilitates the transfer of working fluid into the artificial bladder, generating internal pressure for urine expulsion. Miniaturization of the pump is critical for implantation, as commercially available pumps, despite achieving the required flow rate, are often unsuitable due to size and material constraints. To address this, we developed a compact prototype optimized for consistent and efficient fluid transfer. Computational fluid dynamics (CFD) analysis (Fig. S4) was employed to refine key design parameters, including pump thickness, internal diameter, height, socket length, and tube diameter. The pump was engineered to achieve a flow rate sufficient to expel all stored urine within 10 pumps. In this discharge pressure system, the on/off valve plays a crucial role in regulating the flow of working fluid, ensuring controlled movement between the artificial bladder and the reservoir. The working fluid reservoir was designed to match the bladder's capacity, providing sufficient storage for efficient voiding.

### 3.5 Sensing system for monitoring the urine volume within the artificial bladder

**Fig. 5:**
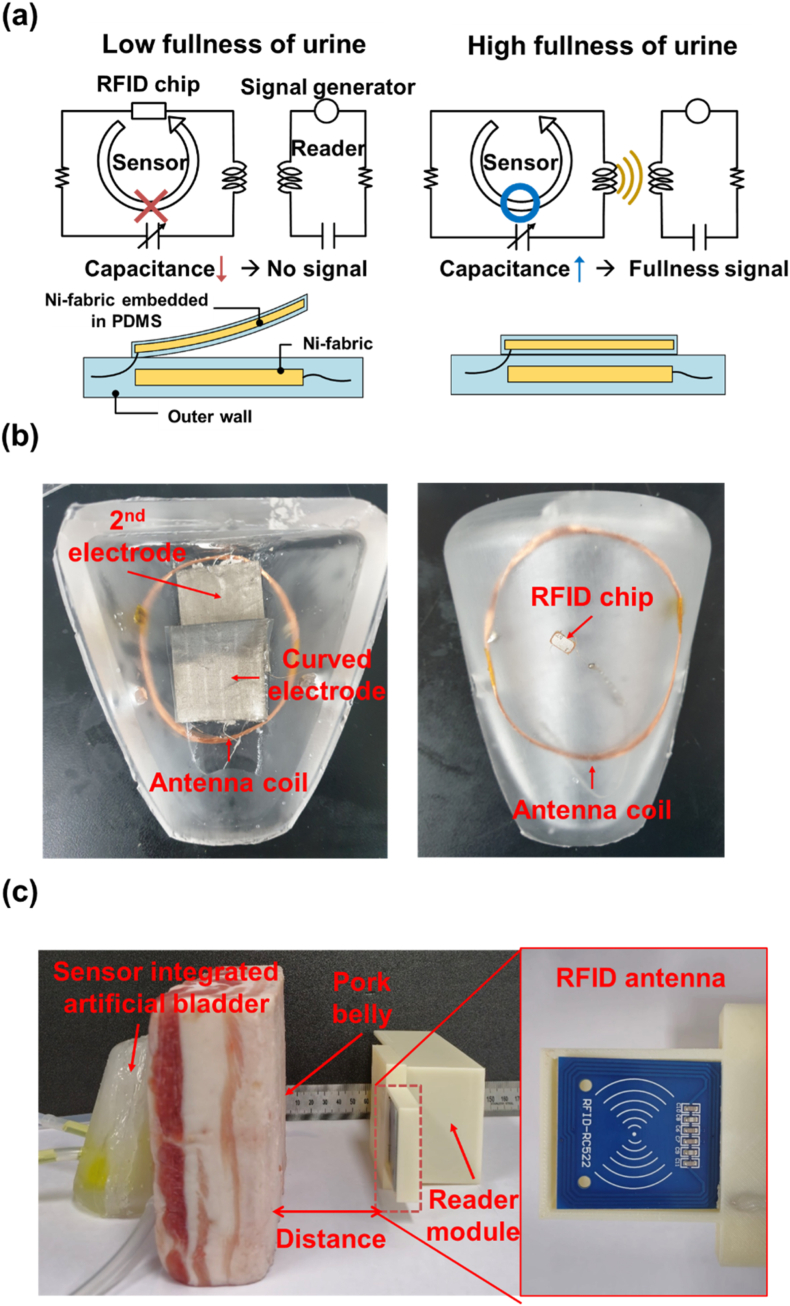
**Working principle and experimental setup of the RFID-based urine volume sensing system.** (a) Schematic of the operating principle of the sensing system that monitors urine volume within the artificial bladder. (b) Optical images of the fabricated sensing system: (left) view of the internal capacitor with curved and fixed electrodes and (right) the integrated RFID chip and antenna coil. (c) Experimental setup to test the communication range of the RFID-based sensor system. The sensor-integrated artificial bladder was encased in pork belly tissue to simulate human abdominal tissue, and the RFID reader was placed at various distances.

Fig. 5a illustrates the working principle of the sensing system for monitoring urine volume, while Fig. 5b presents an optical image of the fabricated sensing system. This study employed RFID technology, a wireless communication method, to detect bladder fullness in the artificial bladder. RFID-based wireless sensors operate without an internal power source, instead receiving power wirelessly from an external reader, thereby eliminating the need for a separate battery [41,42]. The system operates at 13.56 MHz in the high-frequency (HF) band and complies with the ISO/IEC 15693 protocol. This frequency was chosen based on a comparative evaluation of low-frequency (LF), high-frequency (HF), and ultra-high-frequency (UHF) bands. LF tags exhibited limited communication range and required larger antenna dimensions, whereas UHF tags suffered from substantial signal attenuation in biological tissues due to their higher operating frequency. In contrast, the HF band provides an optimal balance between tissue penetration depth and device miniaturization, supporting stable communication through biological tissues up to approximately 5 cm thick. The RFID tag used in this system incorporates a passive integrated circuit designed for ISO 15693, enabling battery-free and reliable wireless operation.

The urine-filling sensor, integrated into the outer wall of the artificial bladder, comprises a capacitor, an RFID chip, and a coil antenna, forming a passive LC resonant circuit (Fig. S5a). The capacitance of the sensor changes in response to deformation of the bladder wall, enabling real-time monitoring of urine volume. The capacitor consists of a curved nickel fabric electrode and a pair of fixed electrodes attached to the inner wall, all passivated with PDMS to ensure electrical insulation and mechanical stability. As the septum membrane deforms with increasing urine volume, the curved electrode is displaced, reducing the distance between electrodes and thereby shifting the resonant frequency of the circuit. This frequency shift is detected by an external HF-band RFID reader, which wirelessly powers the chip and retrieves the corresponding signal. The sensor is embedded within the working fluid partition, specifically along the flat inner wall of the chamber, with an electrode overlapping area of approximately 0.5 cm × 2 cm located 2 cm from the top of the bladder body (Fig. S5b). When deformation exceeds a predefined threshold—corresponding to approximately 75% of the bladder's capacity—the resonant circuit becomes activated, transmitting a detectable RFID signal that indicates bladder fullness. The 75% threshold was selected as an initial proof-of-concept design criterion based on physiological observations that normal urinary desire generally occurs before maximal bladder capacity is reached and that a strong desire to void commonly occurs at approximately 70–80% of bladder capacity [45]. Therefore, the current sensing system was designed to provide a fullness alert near this physiologically relevant range. Below this threshold, the system remains inactive, thereby functioning as an on–off sensor rather than continuously tracking resonance shifts. This threshold-based configuration ensures reliable, battery-free indication of bladder fullness, and the activation level can be tuned by adjusting the position or stiffness of the sensor. While the current system employs a binary, threshold-based sensing approach, this design choice was made deliberately to prioritize robustness, simplicity, and unambiguous user alerts during early-stage validation. This is not a limitation of the sensing mechanism itself, but rather a pragmatic strategy to ensure reliable operation under physiological conditions. Nonetheless, more granular sensing could further enhance user experience and system responsiveness. Transitioning to a multi-threshold or quasi-continuous sensing scheme is technically feasible and aligns with our ongoing development roadmap. Such an enhancement could be realized by integrating multiple capacitive sensors at distinct locations on the bladder wall, employing analog-resolvable RFID ICs capable of frequency interpolation or signal strength analysis, and implementing reader-side algorithms to correlate frequency shifts with urine volume. These improvements primarily involve adjustments to sensor layout and signal processing, without requiring substantial changes to the core implantable hardware. Given the current level of fabrication and integration, this represents a realistic and near-term advancement. After successfully demonstrating urine storage and voiding in the artificial bladder system, we conducted experiments to validate the urine-filling sensor's functionality. In adult males, abdominal tissue thickness is approximately 3 cm [46]. Thus, the urine fullness sensor must maintain a communication range beyond this distance. However, signal transmission can be degraded by the conductivity of blood, bone, and tissue. To address this, as shown in Fig. 5c, the wireless communication range was tested by placing a 5 cm thick piece of pork belly tissue between the sensor and the reader to simulate human conditions. The sensor successfully communicated over a maximum distance of 10 cm. (Movie S3).

Supplementary data related to this article can be found online at https://doi.org/10.1016/j.mtbio.2026.103404

The following are the Supplementary data related to this article:

Multimedia component 5

To further ensure reliable in vivo communication despite potential coil misalignment, we designed the system assuming a wearable patch-type RFID reader to be worn externally on the abdomen, directly above the implanted artificial bladder. This configuration minimizes angular deviation and coil offset, thus maintaining consistent communication. While misalignment may still occur due to body movement or posture, we are considering the future integration of multi-coil or spatially distributed antenna arrays to improve tolerance against angular displacement and extend the effective read range. In addition, the wireless communication stability between the implanted RFID-based urine fullness sensor and the external reader was experimentally validated under various angular and distance misalignments (Fig. S6).

To further validate the performance of the RFID-based bladder fullness sensor, the resonance response was quantitatively evaluated through repeated filling experiments (Fig. S7). In repeated measurements, the sensor exhibited consistent activation behavior with minimal variation in the activation volume, demonstrating good sensing repeatability and reliable threshold detection. Furthermore, angular misalignment and distance variation experiments demonstrated stable wireless operation under up to 30° angular offset and up to 13 cm separation distance, confirming robust sensing performance under practical operating conditions.

Because the sensor operates based on passive LC resonance, capacitance changes induced by septum membrane deformation are reflected immediately in the resonance response. Signal acquisition by the RFID reader occurs within tens of milliseconds, enabling real-time detection of bladder fullness. The resonance characteristics of the RFID-based bladder fullness sensor were evaluated using a function generator and an oscilloscope. An alternating current (AC) signal with a swept frequency was applied to the reader coil through the function generator, while the induced AC voltage amplitude across the sensor coil was monitored via a wired lead connected to the oscilloscope. The resonance frequency was identified as the point of maximum signal amplitude. To assess the effect of positional misalignment, the relative orientation and distance between the antenna and sensor were varied, while maintaining identical excitation conditions and measuring the signal loss. Parasitic capacitance introduced by the temporary wired connection was compensated using calibration values provided by the measurement instruments.

This binary threshold-based sensing approach was adopted in the current implementation to ensure system simplicity, robustness, and minimal power consumption. However, we recognize that this design does not fully capture the gradual and continuous nature of physiological bladder fullness perception. To address this limitation and improve physiological accuracy, future iterations of the system will incorporate multi-level or quasi-continuous sensing strategies. These may include: (i) integration of multiple capacitive sensing units placed at distinct locations within the bladder wall, each activated at specific volume thresholds (e.g., 50%, 75%, and 90% of total capacity) to provide graduated alerts similar to natural urgency progression; (ii) deployment of analog-resolvable RFID integrated circuits capable of detecting finer capacitance variations across the filling range; and (iii) implementation of signal processing algorithms on the reader side to interpolate frequency shifts and estimate bladder volume in a continuous manner, providing real-time feedback rather than discrete threshold alerts.

These enhancements are technically feasible within the existing platform and form part of our ongoing roadmap toward a more physiologically representative and patient-responsive artificial bladder system.

### 3.6 Cytotoxicity of artificial bladder components

Cytotoxicity testing of the Parylene-C coated components was performed in accordance with ISO 10993-5 and ISO 10993-12 standards using the MEM elution method with L-929 fibroblasts at KOREA TESTING LABORATORY (KTL). According to the ISO 10993-5 qualitative morphological grading criteria, the evaluation was performed based on a qualitative assessment rather than a quantitative analysis. Under this standard, samples with cytotoxicity grades of 0-2 on a 0-4 scale are considered non-cytotoxic. As shown in Fig. S8, the cells treated with the extract from the Parylene-C coated sample exhibited morphology and cell density comparable to those of the blank and negative control groups, with no evidence of cell lysis, severe rounding, or growth inhibition. In contrast, the positive control group showed significant cytopathic effects, including extensive cell damage and reduced cell density. The Parylene-C coated components exhibited a cytotoxicity grade of 0, confirming the absence of detectable cytotoxicity and excellent biocompatibility. These results further demonstrate that the conformal Parylene-C coating provides a biologically safe interface suitable for implantation applications.

### 3.7 Evaluation of long-term stability of the artificial bladder via accelerated aging test

To evaluate the long-term stability of the artificial bladder under simulated implantation conditions, an accelerated aging test was conducted at 80 °C and 100% relative humidity for 19 days, simulating one year of physiological exposure. These conditions were selected to replicate the moist internal environment of the bladder while preventing thermal deformation. Independent verification of sealing stability was conducted by an authorized testing institute, and the corresponding certification (Test Report TG-24-282) is available in the Supplementary Data. The functional performance of the artificial bladder was assessed by comparing the infused and discharged volumes of 60 mL of artificial urine before and after aging. Five independent tests were conducted, and in all cases, the input and output volumes remained consistent around the 60 mL target (Fig. S9a). The volume discrepancy, calculated as |Output−Input|/Input×100%, remained below 1.5% across all tests, well within the ±5% acceptance criterion (Fig. S9b). Importantly, no fluid leakage or abnormal discharge was observed during or after the aging process, further confirming the integrity of the system. These results demonstrate that the artificial bladder maintained both mechanical durability and functional performance throughout the accelerated aging test.

### 3.8 In vitro simulation of the urine storage and voiding of the artificial bladder

**Fig. 6:**
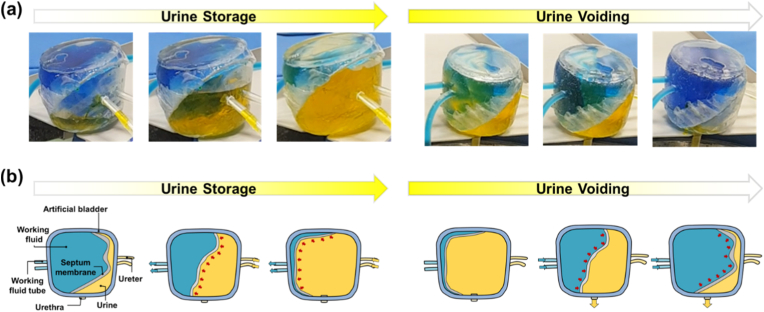
***In vitro* validation of the storage and voiding functions of the artificial bladder.** (a) Optical image showing the storage and voiding phases during urodynamic testing. In the storage phase, urine (yellow liquid) flows into the bladder, causing the septum membrane to move outward and displace the working fluid (blue liquid) into the reservoir. During the voiding phase, external pump pressure draws the working fluid back into the bladder, which pushes the septum membrane inward and expels the stored urine. (b) Schematic illustration of the bladder's internal dynamics during storage and voiding. As urine fills the bladder, the septum membrane moves outward to create space for urine storage. In the voiding phase, the membrane moves back inward under the influence of the working fluid to facilitate urine release. (For interpretation of the references to colour in this figure legend, the reader is referred to the Web version of this article.)

The urine storage and voiding functions of the artificial bladder were evaluated using a clinically employed urodynamic test. During the storage phase, as artificial urine (yellow liquid) flowed into the bladder, a corresponding decrease in the volume of the working fluid reservoir (blue liquid) was observed. This confirmed the outward movement of the septum membrane and an increase in urine storage volume (Fig. 6a, Movie S4). Conversely, during the voiding phase, pressure generated by the external pump was transferred to the working fluid, which moved the septum membrane inward, displacing urine from the storage reservoir (Fig. 6a, Movie S5).

Supplementary data related to this article can be found online at https://doi.org/10.1016/j.mtbio.2026.103404

The following are the Supplementary data related to this article:

Multimedia component 6

Multimedia component 7

The behavior observed during the storage and voiding phases was quantified by tracking pressure changes in the urine reservoir over time. As shown in Table 1, most of the target levels for the prototype were achieved, with key indicators, such as compliance and intra-reservoir pressure, meeting the required standards. The kinematics of the septum membrane, as illustrated in Fig. 6b, were also quantitatively demonstrated. Compliance refers to the bladder's ability to stretch and accommodate increasing urine volumes without causing a significant rise in reservoir pressure. Data from the storage phase indicated that the artificial bladder exhibited excellent compliance, maintaining stable pressure even as it approached full capacity. Pressure stability is a desirable trait in artificial bladder design, as it helps mimic the natural behavior of the human bladder. Pressure records from the voiding phase showed a significant increase in intra-reservoir pressure, confirming the efficient transmission of force from the external pump into the urine reservoir, facilitating effective urine expulsion. Compliance and pressure changes in the urine reservoir are closely correlated and driven by the movement of the septum membrane. This membrane movement enables the reservoir to accommodate incoming urine while ensuring that pressure from the external pump is effectively transmitted to generate voiding pressure, ultimately allowing for efficient urine expulsion.

**Table 1:** Results of the in vitro and in vivo urodynamic tests of the artificial bladder.

	Parameters	Target values^a^	Value	
			*In vitro*	*In vivo*^b^ (*Standard deviation*^c^)
Storage phase	Functional bladder capacity (cc)	200 mL	185	95 (5)
	Maximum pressure (cmH_2_O)	<25 cmH_2_O.	8	39.94 (7.15)
	Mean compliance (mL/cmH_2_O)^d^	12.5∼30 mL/cmH_2_O	23.1	18.02 (1.86)
Voiding phase	Effective voiding volume (cc)	N/A	165	89 (3)
	Peak pressure (cmH_2_O)	40∼60 cmH_2_O	98.1	119.98 (2.11)
	Mean pressure (cmH_2_O)	40∼60 cmH_2_O	N/A	63.07 (9.09)
	Pump mean pressure (cmH_2_O)	N/A	N/A	74.29
	Residual volume	<50 mL	20	5 (1.97)
	Voiding efficacy (Effective voiding volume/Functional bladder capacity)	>0.9	0.89	0.94 (0.02)

a The design goals of the artificial bladder are based on normal physiological functions.

b Measured by applying a threshold valve (set at 50 cm H_2_O) to the Foley catheter.

c The standard deviation data for the in vivo experiments are as follows. Repeated experiments were conducted using the same pig body, with a total of three in vivo experimental cycles. The in vitro experiment was conducted at the early stage of this study to evaluate whether the storage and voiding functions of the artificial bladder prototype were functioning properly. Since the functionality of the artificial bladder was sufficiently confirmed through a single experiment, no further in vitro testing was conducted.

d The compliance C is the volume change (ΔV) over the pressure change (ΔP). Table 1 details the compliance C_1_ for segment 1, derived from points S_1_ to M. The compliance C_2_ for segment 2, derived from points S_1_ to S_4_, is 7.38 cc/cm H_2_O, The compliance C3 for segment 3, derived from points S_4_ to V_4_, is 2.83 cc/cm H_2_O. Fig. 6 shows the constants S_1_, S_4_, V_4_, and M.

### 3.9 Implantation of the artificial bladder into a pig model

**Fig. 7:**
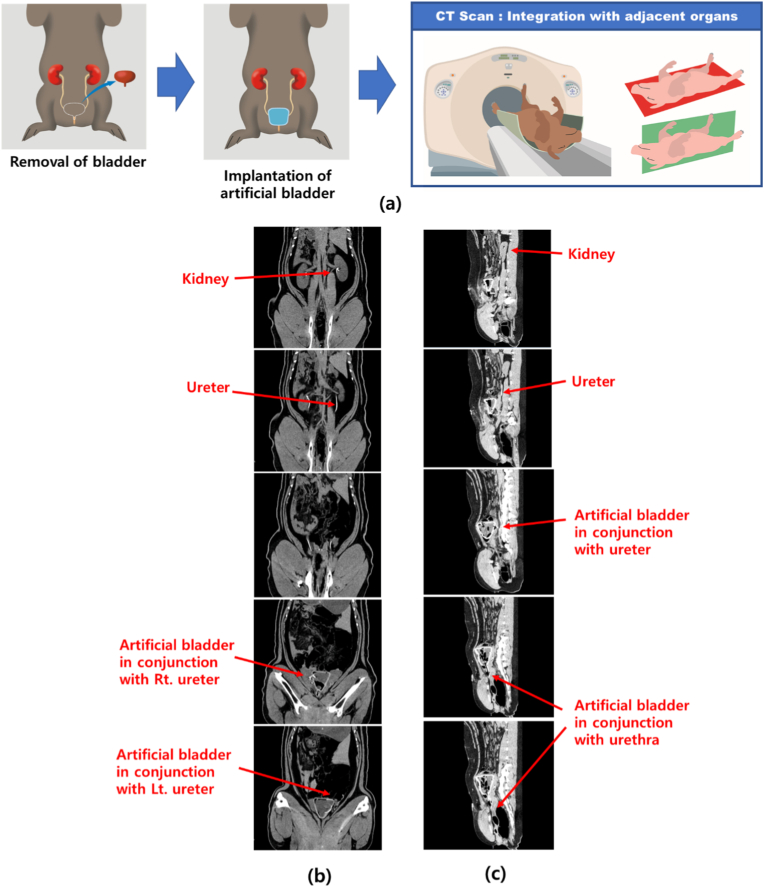
**Surgical procedure and CT imaging of artificial bladder implantation.** (a) Schematic representation of the surgical steps: removal of the native bladder, implantation of the artificial bladder, followed by CT imaging to assess anatomical integration with adjacent organs. Computed tomography (CT) scan images illustrating the urinary tract after the implantation of an artificial bladder in a mini pig model. (b) Coronal view showing the kidney, ureter, and artificial bladder connected to the right and left ureters. (c) Sagittal view demonstrating the kidney, ureter, and artificial bladder integrated with the urethra. Post-operative imaging confirmed effective urine drainage with no significant complications, and the contrast medium showed no evidence of leakage, indicating functional integrity and proper connection within the urinary system.

The mini pigs that underwent artificial bladder implantation successfully achieved urine drainage, with no significant complications observed during the postoperative period. Fig. 7a illustrates the sequential steps of the surgery, starting with the excision of the native bladder, followed by the placement of the artificial bladder, and concluding with a computed tomography (CT) scan to assess anatomical integration with adjacent organs. Fig. 7b and c provide detailed coronal and sagittal CT images, respectively. In the coronal view, the kidneys and ureters are clearly visible, confirming the successful attachment of the artificial bladder to both the right and left ureters. The sagittal view further supports this integration, demonstrating the alignment of the artificial bladder with the ureters and its connection to the urethra. These imaging findings confirm the anatomical feasibility and proper positioning of the artificial bladder, indicating a successful surgical outcome and effective integration within the urinary tract system.

**Fig. 8:**
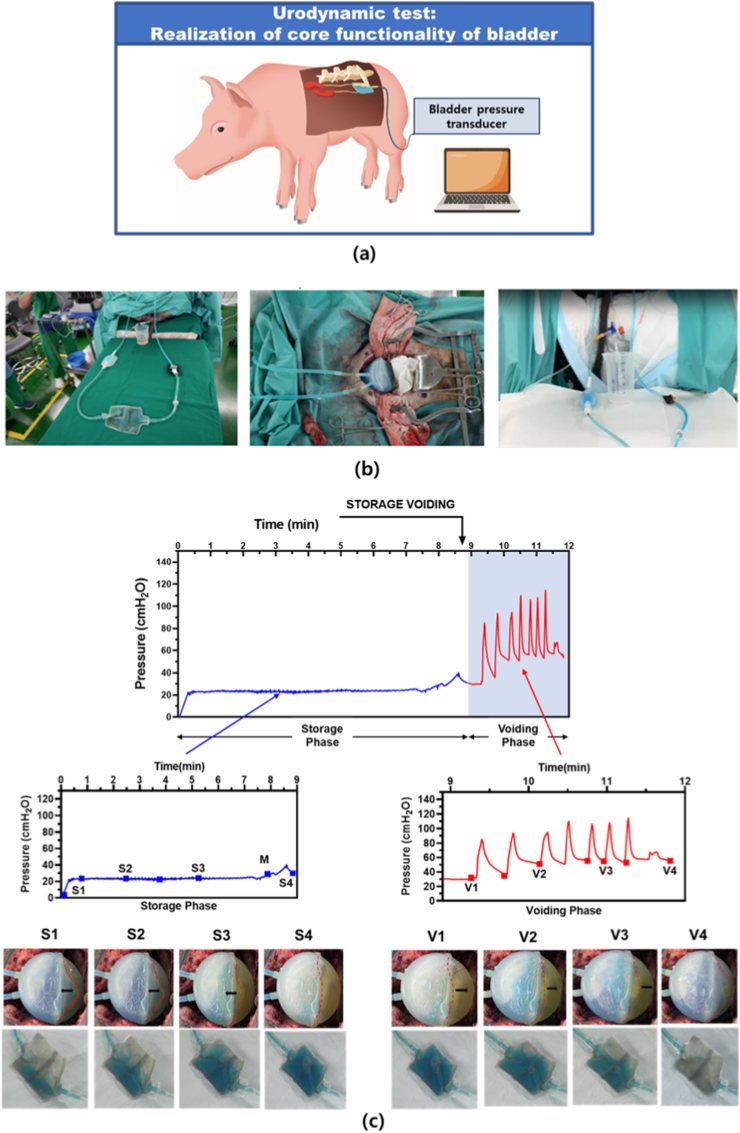
***In vivo* urodynamic evaluation of the artificial bladder.** (a) Schematic illustration of the urodynamic test performed six weeks post-implantation. (b) Photo of experimental set-up for urodynamic test showing the implanted artificial bladder connected to a voiding pressure control system. (c) Urodynamic test results in an anesthetized pig. The upper graph presents the pressure profile during the storage (S1∼S4) and voiding (V1∼V4) phases. The lower panels depict the septum membrane dynamics and working fluid movement, demonstrating effective urine storage and voiding. S1 is the point where the bladder internal pressure of the storage phase stabilizes, and S4 is the end point of the storage phase. S2 and S3 are equidistant points between S1 and S4, divided at equal intervals based on the stored urine volume. The transition from V1 to V2 is defined as the point following two manual pump actuations (compressions). The transition from V2 to V3 is defined by three pump actuations. The transition from V3 to V4 entails three manual actuations, with V4 defined as the final pressure point where the voiding process is fully completed upon the last actuation.

A urodynamic evaluation of the artificial bladder was conducted six weeks after implantation, as illustrated in Fig. 8a. Fig. 8b presents a photograph of the experimental setup for the evaluation. A suprapubic midline laparotomy was performed to expose the implanted artificial bladder, and the inlet and outlet tubes for the working fluid were connected to the voiding pressure control system. To record pressure changes and infuse saline into the urine storage partition, three-way stopcock tubing and a threshold valve (set at 50 cm H_2_O) were inserted into a Foley catheter placed in the artificial bladder. The tubing was then connected to a pressure transducer and syringe pump. Fig. 8c presents the results of in vivo urodynamic studies. Cystometrographic activity and artificial bladder function were recorded during continuous saline infusion in an anesthetized pig with the implanted artificial bladder. The upper panel displays the pressure profile during the storage (S1∼S4) and voiding (V1∼V4) phases. The results demonstrated good compliance of the storage partition (18.02 mL/cmH_2_O) and the ability of the septum membrane to expand, enabling urine storage (95 mL) without significant leakage or pressure fluctuations. The lower panels illustrate the dynamics of the septum membrane and working fluid. During the storage phase (S1∼S4), the septum membrane shifts leftward as urine fills the bladder, while the working fluid gradually refills the reservoir. In the voiding phase (V1∼V4), the working fluid flows back into the artificial bladder, pushing the septum membrane rightward, which empties the reservoir and effectively expels urine.

Movie S6 provides a visual demonstration of the artificial bladder's storage and voiding functions during in vivo evaluation. As urine fills the artificial bladder, the septum membrane moves, displacing the working fluid into the reservoir. Once the bladder reaches full capacity, the external manual pump is activated, forcing the working fluid back into the working fluid partition, thereby expelling the stored urine efficiently. This video further supports the functional validation of the artificial bladder, reinforcing the results presented in Fig. 8.

Supplementary data related to this article can be found online at https://doi.org/10.1016/j.mtbio.2026.103404

The following are the Supplementary data related to this article:

Multimedia component 8

These results indicate that the artificial bladder can function similarly to a natural bladder in terms of urine storage. Furthermore, the stored urine was successfully emptied using the voiding pressure control system, demonstrating the artificial bladder's ability to effectively expel urine. The external pump, responsible for generating voiding pressure, efficiently transmitted pressure within the urine storage partition during the voiding phase (peak: 120 mmHg; mean: 63 mmHg), ensuring a smooth and consistent urine flow through the urethra. The successful operation of the pump underscores the importance of a well-designed external control system for artificial bladders. Additionally, these results suggest that external pumps could serve as a viable option for future artificial bladder designs, providing greater control and customization over urination patterns. Table 1 summarizes the results of the urodynamic studies.

In the large animal model (mini pig), manual assistance by the research team was required to induce urination, as voluntary control of the urethral sphincter is not achievable in pigs. However, continuous monitoring and manual voiding over the entire 6-week experimental period was not practically feasible. To address this limitation, a Foley catheter was employed to enable continuous urine drainage, allowing the bladder to be emptied regularly without relying on sensor-based fullness detection. Consequently, implantation of the RFID-based sensor was not included in this phase of the study. In future human applications, however, the integrated bladder fullness sensor is expected to play a critical role by providing timely alerts for voluntary voiding, thereby supporting user-directed or semi-autonomous control of urination.

To clarify the urine drainage method during the six-week in vivo period, a Foley catheter was maintained for three primary reasons. First, it supported tissue healing and stabilized the anastomosis between the artificial bladder and urethra. Upon explantation after six weeks, secure urethral connections were confirmed. Second, since pigs could not voluntarily activate the voiding mechanism, and frequent manual operation during conscious periods was impractical, catheterization enabled consistent drainage while maintaining animal welfare. Third, physiological urination requires coordinated bladder pressure increase and voluntary urethral sphincter relaxation (detrusor-sphincter synergy), which is unachievable in experimental animals due to their inability to consciously coordinate sphincter control with the artificial system.

The Foley catheter was connected to a threshold valve set at 50 cm H_2_O to enable pressure-driven drainage at predetermined filling levels. Additionally, dedicated urodynamic evaluations at 6 weeks demonstrated functional performance under controlled conditions, achieving 95 mL storage capacity and 94% voiding efficiency when the pump system was activated.

Importantly, these limitations are specific to the animal model. In human applications, conscious control over pump activation and voluntary sphincter relaxation would permit natural coordinated voiding, and the artificial bladder's functional mechanism remains fully applicable.

To further clarify the role of the urethral sphincters in relation to the artificial bladder system, the following anatomical and functional considerations are noted. Anatomically, the urethral sphincter are located within the urethra, distal to the bladder. In our implantation model, the artificial bladder was surgically anastomosed to the urethra while preserving the native sphincter structures. The design of the artificial bladder does not interfere with their anatomical positioning or function.

### 3.10 Investigation of the safety and structural integrity of the artificial bladder up to six weeks post-transplantation

The comparison of blood chemistry (including renal function, liver function, and electrolytes), complete blood count (CBC), weight, and general condition of the pigs before and after surgery remained favorable for up to six weeks post-transplantation (Table 2). Renal function, as indicated by blood urea nitrogen (BUN) and creatinine levels, was well-maintained in tests conducted six weeks after artificial bladder transplantation. Additionally, hematological parameters, including hemoglobin levels, electrolytes, and hepatobiliary function, remained stable without deterioration throughout the post-transplantation period.

**Table 2:** Comparison of hematological and blood chemistry tests before and after artificial bladder implantation.

		Before surgery	1 week	2 weeks	4 weeks	6 weeks
Hematology	WBC (×10^3^ cells/uL)	9.64	15.26	11.19	14.8	12.48
	RBC (×10^6^ cells/uL)	4.88	4.04	3.86	3.68	3.13
	HGB (g/dL)	10.5	8.8	8.4	7.5	6.6
	HCT (%)	30.6	24.9	24.5	23	20.2
	PLT (×10^3^ cells/uL)	271	354	426	423	632
Renal function	BUN (mg/dL)	7.2	12.1	13.5	9.8	9.7
	Cr. (mg/dL)	1.5	1.811	1.91	1.143	1.608
Nutrition status	TP (g/dL)	7.5	7.4	7.1	7.4	8.4
	Alb (g/dL)	4.902	4.419	4.301	4.336	4.388
Hepatobiliary function	TB(mg/dL)	0.024	0.319	0.016	0.044	0.015
	AST (U/L)	19	30	17	16	18
	ALT (U/L)	22	29	21	19	20
	ALP (U/L)	148	101	131	137	69
Electrolytes	Na (mmol/L)	143.5	141.7	142.7	140.9	142.9
	K (mmol/L)	4.8	5.1	4.2	4.4	4.8
	CL (mmol/L)	102.6	101.8	101.9	100.3	98.2

WBC, white blood cell count; RBC: red blood cell count; HGB: hemoglobin, HCT: hematocrit, PLT: platelet count; BUN: blood urea nitrogen, Cr: creatinine, TP: total protein, Alb: albumin, TB: total bilirubin, AST: aspartate aminotransferase, ALT: alanine aminotransferase, ALP: alkaline phosphatase, Na: sodium, K: potassium, Cl: chloride.

**Fig. 9:**
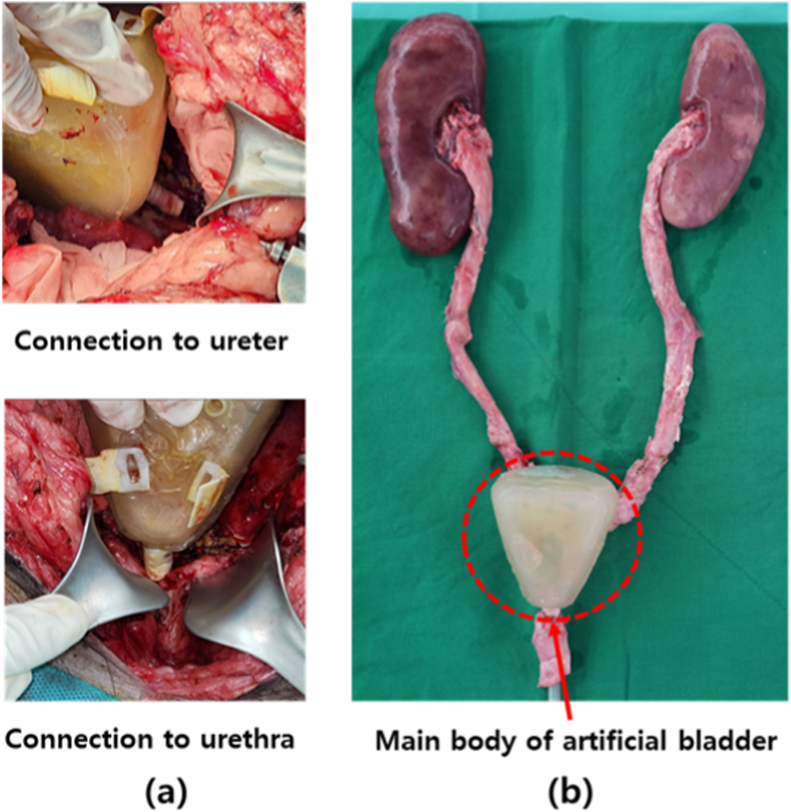
**Surgical connection and post-implantation integrity of the artificial bladder.** (a) Intraoperative images showing the surgical connection of the artificial bladder to the ureters (top) and urethra (bottom) 6 weeks after implantation. (b) Excised specimen displaying the main body of the artificial bladder (highlighted with a red dashed circle) successfully integrated with the kidneys, ureters, and urethra, demonstrating its anatomical feasibility and secure attachment. (For interpretation of the references to colour in this figure legend, the reader is referred to the Web version of this article.)

Throughout the six-week follow-up period, both feeding behavior and activity levels remained normal. Additionally, the connections between the artificial bladder, ureters, and urethra remained secure (Fig. 9a). The implanted artificial bladder maintained its structural integrity, showing no cracks or damage. Fig. 9b presents the overall structure of the implanted artificial bladder system, demonstrating its seamless integration with the kidneys, ureters, and urethra. The successful anatomical reconstruction highlights the feasibility of the artificial bladder in restoring normal urinary function.

To minimize fibrotic tissue response and calcification, both internal and external surfaces of the artificial bladder were conformally coated with a 3 μm-thick Parylene-C layer. This biocompatible coating is known to reduce tissue adhesion, chronic inflammation, and mineral deposition. Fig. 9b shows that the explanted artificial bladder retained its structural integrity and anatomical connectivity with the ureters and urethra, with no visible signs of fibrous tissue overgrowth or encapsulation. Furthermore, Alizarin Red S staining and quantitative absorbance analysis revealed a significant reduction in calcium deposition in the urine compartment of Parylene-C coated bladders compared to non-coated controls, while the working fluid compartment showed negligible calcification in both groups (Fig. S10). This result not only demonstrates the anti-fouling effect of the Parylene-C coating in direct contact with urine but also confirms the effectiveness of the septum membrane in preventing fluid crossover between the two compartments.

## 4 Discussion

The development and evaluation of an artificial bladder capable of performing the primary physiological functions of a natural bladder represent a significant advancement in biomedical engineering. The proposed artificial bladder is designed to replicate essential bladder functions, namely urine storage and voiding, while maintaining a stable connection with adjacent organ systems under physiological conditions. This device mimics the dynamic processes of the natural bladder through the innovative use of partitioned spaces, separated by a septum membrane, demonstrating effective urine storage and controlled voiding capabilities.

The fabrication process incorporates advanced techniques, such as replica molding and 3D printing, ensuring precise construction with high-quality materials. The silicone-based main body, combined with a glass fiber-embedded PDMS septum membrane, provides structural integrity and flexibility. Additionally, the Parylene-C coating enhances biocompatibility and mechanical stability, which are critical for long-term in vivo durability. Cytotoxicity tests confirmed that these materials are biocompatible and safe for direct contact with human tissues, an essential factor for the overall safety and efficacy of the device.

The storage function relies on the engineered septum membrane, which separates urine from the working fluid. This membrane is sufficiently flexible to deform in response to urine volume changes while minimizing elastic recovery force to prevent negative pressure formation. The glass fiber reinforcement within the PDMS septum reduces elastic recovery force and improves durability. In vitro tests demonstrated that the artificial bladder could store desired amount of urine while maintaining a stable intravesical pressure, successfully mimicking the mechanical properties of a natural bladder. These findings indicate that the artificial bladder can effectively accommodate varying urine volumes. The voiding function is managed by a manual pump system, which, along with an on/off valve and working fluid reservoir, allows for precise voiding control. The innovative design generates voiding pressure by expanding the working fluid partition, effectively transporting fluid into the bladder and ensuring efficient urine expulsion, as confirmed in both in vitro and in vivo studies.

The manual pump, a key component enabling controlled voiding, was fabricated using Dragon Skin-30 silicone rather than the more commonly used PDMS. This material was selected for its higher elasticity (Shore A 30) and superior mechanical resilience under repeated compression cycles, which are critical for long-term manual operation. Dragon Skin-30 passed ISO 10993-5 cytotoxicity testing, confirming its non-cytotoxic nature. While this supports its basic biocompatibility, we acknowledge that additional in vivo studies—such as those evaluating chronic inflammation, fibrosis, and tissue integration—are required to determine its suitability for short- or long-term implantation (Table S1).

To enhance usability, an RFID-based wireless sensor was incorporated to detect bladder fullness. This noninvasive sensor operates without an internal power source, instead drawing energy wirelessly from an external reader, eliminating the need for batteries [[47], [48], [49], [50], [51]]. Importantly, the RFID-based urine fullness sensor serves as an auxiliary monitoring component rather than a safety-critical component required for basic bladder operation. The urine storage and manual voiding functions of the artificial bladder can be maintained independently of the sensing system. Therefore, temporary sensor malfunction does not prevent fundamental bladder operation. To further improve sensing reliability, future versions of the system may incorporate redundant multi-sensor architectures and additional signal validation algorithms capable of maintaining bladder fullness monitoring even in the event of individual sensor failure. Furthermore, wireless communication stability was experimentally evaluated under angular and distance misalignment conditions that simulate positional variations associated with posture changes and body movement. Although stable signal detection was confirmed under moderate misalignment conditions, additional studies investigating long-term sensor reliability, dynamic body motion, and unexpected sensing failure scenarios will be required prior to clinical translation. The present study employed a binary threshold-based sensing strategy rather than continuous bladder volume estimation. Future developments will focus on multi-threshold sensing architectures and continuous volume estimation algorithms capable of providing more physiologically representative bladder fullness information. The sensor provided timely alerts when the bladder reached 75% capacity, enhancing patient comfort and enabling more effective management. Although the focus was primarily on storage and voiding, the integration of this sensor increases the versatility of the artificial bladder for future applications.

In vitro simulations confirmed that the artificial bladder effectively performed both storage and voiding functions. During the storage phase, the fluid reservoir volume decreased as urine was stored, whereas during the voiding phase, the septum membrane moved to facilitate urine expulsion. These simulations validated the functional design, demonstrating stable urine storage of up to 180 mL and consistent compliance. Additionally, pressure flow studies confirmed that externally generated pump pressure effectively increased intra-bladder pressure, facilitating efficient urine voiding. In experiments involving open beakers, care was taken to ensure that at any given time, either the urine chamber or the working fluid chamber was sealed using on/off and check valves. During the urine storage phase, only the working fluid return valve was open, while all other valves remained closed (Fig. S11). In this configuration, the urine storage chamber was effectively sealed, and internal pressure was balanced through its coupling with the open working fluid chamber. During the voiding phase, the working fluid valve was closed and only the urine outlet valve was opened, reversing the configuration. The working fluid chamber remained sealed, and the urine chamber operated against it. Throughout both phases, the system was fully governed by the valve mechanism, and the functional fluid loop maintained the behavior of a closed hydraulic system.

An in vivo study in a mini pig model further demonstrated the functionality of the artificial bladder, with successful urine drainage and no significant postsurgical complications. Imaging and renal function tests conducted over a six-week period confirmed proper integration of the artificial bladder with the urinary tract. Urodynamic studies revealed good compliance and effective urine storage without leakage. The ability to empty stored urine using the pressure control system validated the operational effectiveness of the bladder, emphasizing the importance of a well-designed external control system. The artificial bladder maintained renal function and seamless integration with the kidneys, ureters, and urethra throughout the post-transplantation period, suggesting its suitability for long-term use. The favorable biocompatibility profile, as evidenced by stable blood chemistry, weight, and overall condition, further supports its potential clinical applications.

While the present study demonstrated favorable functional stability and subchronic biological safety through accelerated aging tests and 6-week implantation in a mini-pig model, these evaluations do not fully represent the chronic biological environment encountered by long-term urinary implants. Clinically implanted urinary devices are continuously exposed to urine and may gradually develop complications such as protein adsorption, bacterial biofilm formation, calcification, chronic inflammation, and foreign-body responses during prolonged implantation periods.

The major materials used in this system, including silicone-based elastomers and Parylene-C coating, were selected based on their established biocompatibility and widespread use in implantable medical devices. Silicone-based elastomers are well known for their favorable mechanical compliance, chemical inertness, and low tissue irritation, which may help minimize friction-induced tissue damage at tissue-device interfaces [52,53]. In addition, the smooth and chemically stable surface of Parylene-C may reduce mechanical irritation while suppressing mineral deposition and surface fouling under urinary conditions [[54], [55], [56], [57]]. In particular, Fig. S10 demonstrated that the Parylene-C coating effectively suppressed calcification on the urine-contacting surfaces of the artificial bladder during the implantation period, suggesting its potential protective anti-fouling effect under physiological conditions.

Encrustation on the one-way check valve may additionally affect long-term valve performance because mineral deposition and biofilm-associated crystal growth can gradually accumulate on urine-contacting surfaces. Additional material and coating strategies may further improve resistance against encrustation-related valve dysfunction. Representative approaches include grafting of hydrophilic polymers such as polyethylene glycol (PEG) or poly(acrylic acid) to suppress protein adsorption and crystal nucleation, hydrogel-based anti-fouling coatings to generate highly hydrated interfaces, and zwitterionic surface modifications to minimize nonspecific biomolecular adhesion. Antibacterial coatings may additionally help suppress biofilm formation, which is closely associated with urinary encrustation and mineral accumulation. These surface engineering approaches may contribute to maintaining stable long-term valve operation by reducing urinary precipitate deposition on the valve surface [[58], [59], [60]].

Nevertheless, chronic urinary exposure may still induce gradual surface degradation, mineral deposition, or chronic foreign-body reactions over time. Accordingly, future studies will focus on extended urine-immersion testing, long-term cyclic filling–voiding evaluations, chronic large-animal implantation, detailed histological analysis of tissue-device interfaces, biofilm formation, calcification, and material degradation. These investigations will be critical for establishing the long-term durability, chronic biological stability, and clinical feasibility of the artificial bladder system.

The native ureters were preserved in this system, and urine transport into the artificial bladder is therefore expected to remain dependent on physiological ureteral peristalsis. The low-pressure storage state of the artificial bladder may provide a favorable condition for antegrade urine inflow, as supported by stable renal function, absence of hydronephrosis, and preserved urinary tract continuity. However, ureteral peristalsis was not directly measured in this study. Future studies should evaluate ureteral transport efficiency, upper tract pressure, and reflux risk during storage and voiding.

The demonstrated sealing performance and mechanical stability of the artificial bladder system support its suitability for long-term intra-abdominal implantation. Accelerated aging and repetitive mechanical loading tests confirmed watertight integrity without signs of leakage, delamination, or structural failure. The PDMS–Ecoflex composite structure, along with the dual critical junctions—between the artificial bladder and Gore-Tex, and between Gore-Tex and native tissues (ureters and urethra)—effectively maintained luminal patency and water-tightness throughout the six-week in vivo study. No urine leakage, obstruction, or peritoneal inflammation was observed, and the animals remained physiologically stable without adverse events. These findings indicate that the current sealing approach is capable of withstanding the mechanical and biological stresses associated with implantation. While further optimization of interface design may improve safety margins, the current strategy already offers a strong foundation for clinical translation.

For human implantation of the artificial bladder system, The working fluid reservoir will be located within the subcutaneous fat layer of the abdomen. Since abdominal fat is of low density and has inherent expansibility, creating sufficient space for the reservoir by removing some existing fat tissue should not pose a problem. The artificial bladder itself will be placed within the abdominal cavity, where the native bladder was originally located. Importantly, the artificial bladder and the working fluid reservoir will be positioned in separate anatomical compartments, divided by the abdominal muscle layer (Fig. S12).

The clinical goal of the artificial bladder system differs fundamentally from that of external urinary diversion, such as urostomy. Although a ureter-connected urostomy conduit may provide a low-cost method of urinary diversion, it requires continuous external urine collection using an appliance or urine bag, which may compromise quality of life due to body image concerns, appliance management, leakage, skin irritation, odor, infection risk, and lifelong external care. In contrast, the artificial bladder is designed to function as an internal, body-integrated reservoir that stores urine at low pressure and allows controlled emptying without continuous external urine collection. Therefore, its value lies not simply in urinary drainage, but in restoring a more physiological form of urine storage and controlled voiding.

Intra-abdominal pressure (IAP) is an important physiological factor that may influence artificial bladder mechanics. In our system, increased IAP is expected to be transmitted simultaneously to the artificial bladder, ureters, urethra, and hydraulic components, creating a relatively uniform pressure equilibrium rather than a localized pressure gradient. Given the relatively low range of physiological IAP and the high mechanical stiffness of the PDMS–Ecoflex composite outer wall, normal abdominal pressure is unlikely to substantially deform the artificial bladder or impair its internal compartments [61]. In addition, the preserved native ureters and urethra are expected to maintain urine flow through physiological peristalsis and patency under normal IAP conditions. However, dynamic IAP changes during coughing, straining, exercise, or positional changes were not quantitatively evaluated in this study and should be investigated in future studies.

The manual pump and the on/off valve are designed to be implanted in the scrotal pouch, following current clinical practices established in artificial penile prosthesis implantation (Fig. S13). This location provides convenient manual access and allows for discreet user control. In the proposed system, the manual pump and the on/off valve are positioned on the left and right sides of the scrotum, respectively, enabling independent and intuitive operation. In practical physiological environments, transient increases in intra-abdominal pressure (IAP) are uniformly transmitted to both the urinary bladder and the surrounding urinary tract, resulting in near-equilibrium pressure across the system. Consequently, abdominal compression does not induce a pressure differential sufficient to cause backflow, as both the storage chamber and outlet pathway experience the same pressure load. This principle parallels native bladder physiology, in which the ureterovesical junction and the compliant bladder wall prevent reflux during elevated abdominal strain. In our artificial system, the same condition is preserved, and, if necessary, a one-way check valve can be incorporated at the ureter–reservoir interface to provide mechanical isolation against transient pressure fluctuations. To prevent reflux of urine toward the kidneys caused by potential back pressure from the reservoir, a one-way check valve is planned to be installed at the junction between the kidneys and the artificial bladder in future human applications [62,63].

Another important consideration for future clinical translation is the potential risk of obstruction at the ureter–artificial bladder junction if an anti-reflux one-way valve is incorporated. Urinary stones originating from the renal pelvis or ureter could theoretically obstruct the valve, impair antegrade urine drainage, and lead to urinary tract obstruction and renal function deterioration. Future anti-reflux systems should therefore maintain a wide, low-resistance antegrade flow pathway and avoid narrow or easily occluded valve structures. Possible modifications include smooth non-stenotic connector geometry, low-opening-pressure flap-type or duckbill-type valves. Clinically, stone prevention will also be important through adequate hydration, dietary modification, and, when indicated, preventive therapy such as potassium citrate.

The urodynamic evaluation revealed peak voiding pressures exceeding typical physiological ranges due to manual pump activation. Although this pressure was effective for bladder emptying, excessive intravesical pressure may increase the risk of vesicoureteral reflux and upper urinary tract stress. Therefore, future designs will require more precise pressure-control strategies, including pump downsizing, stroke limitation, adjustable threshold valves, and anti-reflux mechanisms, to minimize excessive pressure transmission while preserving effective voiding. In addition, although each animal's preoperative values were used as baseline controls for longitudinal comparison, the absence of a sham-operated control group remains a limitation of this study. The present results support preliminary feasibility and short-term safety; however, this study design cannot fully distinguish device-specific effects from surgical or perioperative factors. Future studies should include sham-operated controls, multiple animals, predefined renal safety endpoints, and validation of pressure-control modifications to more rigorously evaluate device-specific safety, vesicoureteral reflux risk, and upper urinary tract function.

A limitation of this study is that the in vivo results were presented as representative proof-of-concept data rather than statistically powered validation data. Therefore, there are limitations in presenting formal statistical analysis and biological reproducibility. Future studies with larger animal cohorts, standardized perioperative protocols, appropriate control groups, predefined endpoints, and formal statistical analyses will be required to confirm biological reproducibility, renal safety, long-term durability, and clinical translatability.

This study presents a comprehensive evaluation of an artificial bladder that successfully replicates the primary functions of a natural bladder. With robust in vitro and in vivo validations, the artificial bladder shows significant potential for clinical applications, marking a milestone in bladder prosthetics. To the best of our knowledge, this is the first study to demonstrate these core functions in a living organism and to offer a promising solution for patients requiring bladder replacement or restoration. This advancement underscores the potential for future innovations in bladder prosthetics and paves the way for improved treatments for bladder dysfunction.

## 5 Conclusion

This study presents a bioengineered artificial bladder that successfully restores key bladder functions, including storage, sensing, and controlled voiding. Using a PDMS-Ecoflex composite with a Parylene-C coating, the device ensures mechanical flexibility and biocompatibility, while a glass fiber-reinforced septum membrane stabilizes intra-bladder pressure. A battery-free RFID sensor enables real-time urine volume monitoring, enhancing usability. In vitro and in vivo (mini-pig, 6 weeks) studies demonstrated the artificial bladder's functionality, biocompatibility, and structural integrity. The device maintained stable pressure during storage, generated sufficient voiding pressure, and showed effective urine drainage without significant complications. The robustness and sealing reliability of the device were further supported by independent verification conducted by an accredited medical testing foundation (Test Report TG-24-282), reinforcing the technological readiness of the system for future translational and clinical studies. These findings highlight the potential of biomaterial-based artificial organs for clinical translation. Future research will focus on optimizing material properties and enhancing long-term functionality for bladder prosthesis applications.

## CRediT authorship contribution statement

**Gyujun Choi:** Data curation, Investigation, Validation, Visualization, Writing – original draft, Writing – review & editing. **Jinho Kim:** Conceptualization, Data curation, Methodology, Validation, Writing – original draft, Writing – review & editing. **Won-Gun Koh:** Conceptualization, Data curation, Methodology, Validation, Writing – original draft, Writing – review & editing. **Jaehoon Jung:** Data curation, Methodology, Resources, Writing – review & editing. **Ryounghoon Jeon:** Investigation, Resources. **Jung Bae Seong:** Investigation, Resources. **Youngjeon Lee:** Investigation, Resources. **Taehyeon Kim:** Investigation, Validation, Visualization. **Wonkeun Park:** Investigation, Visualization. **Jeonghyeop Son:** Investigation, Visualization. **Bowoong Heo:** Investigation, Visualization. **Soojin Park:** Investigation, Validation, Visualization. **Jongwon Kim:** Investigation, Validation, Visualization. **Jeong-Mu Cheon:** Investigation, Resources, Validation. **JunJie Piao:** Investigation. **Yun-Hee Lee:** Project administration, Visualization. **Jongbaeg Kim:** Conceptualization, Data curation, Methodology, Supervision, Validation, Writing – original draft, Writing – review & editing. **U-Syn Ha:** Conceptualization, Data curation, Methodology, Supervision, Validation, Writing – original draft, Writing – review & editing.

## Declaration of competing interest

The authors declare that they have no known competing financial interests or personal relationships that could have appeared to influence the work reported in this paper.

## Data Availability

Data will be made available on request.

## Appendix A Supplementary data

Supplementary Data contains 15 figures, 6 movies and Official test certificate (Issue No. TG-24-282, Daegu-Gyeongbuk Medical Innovation Foundation, issued May 23, 2025).
