# Supplementary material for: Preclinical development of engineered biomaterial-based artificial bladder demonstrating core functions in a large-animal orthotopic model
Source: Mater Today Bio. 2026 Jun 29;39:103404. doi: 10.1016/j.mtbio.2026.103404 (PMC13355429; doi:10.1016/j.mtbio.2026.103404)
Supplement: Multimedia component 1 [file mmc1.docx]

**Preclinical Development of Engineered Biomaterial-Based Artificial Bladder Demonstrating Core Functions in a Large-Animal Orthotopic Model**

Gyujun Choi^1†^, Jinho Kim^3†^, Won-Gun Koh^4†^, Jaehoon Jung^5^, Ryounghoon Jeon^5^, Jung Bae Seong^6^, Youngjeon Lee^6^, Taehyeon Kim^4^, Wonkeun Park^1^, Jeonghyeop Son^1^, Bowoong Heo^1^, Soojin Park^3^, Jongwon Kim^3^, Jeong-Mu Cheon^5^, JunJie Piao^2^, Yun-Hee Lee^2^, *Jongbaeg Kim*^1*^, and *U-Syn Ha*^2*^

^1^ School of Mechanical Engineering, Yonsei University, 50 Yonsei-ro, Seodaemun-gu, Seoul 03722, Republic of Korea

^2^ Department of Urology, Seoul St. Mary’s Hospital, College of Medicine, The Catholic University of Korea, 222 Banpo-daero, Seocho-gu, Seoul 06591, Republic of Korea

^3^ School of Mechanical Engineering, Yeungnam University, 280 Daehak-ro, Gyeongsan 38541, Republic of Korea

^4^ Department of Chemical and Biomolecular Engineering, Yonsei University, 50 Yonsei-ro, Seodaemun-gu, Seoul 03722, Republic of Korea

^5^ Daegu-Gyeongbuk Medical Innovation Foundation, 88 Dongnae-ro, Dong-gu, Daegu 41061, Republic of Korea

^6^National Primate Research Center, Korea Research Institute of Bioscience and Biotechnology, 30 Yeongudanji-ro, Ochang-eup, Cheongwon-gu, Cheongju-si, Chungcheongbuk-do 28116, Republic of Korea.

†**These authors contributed equally to this work.**

Prof. Jongbaeg Kim

School of Mechanical Engineering, Yonsei University, 50 Yonsei-ro, Seodaemun-gu, Seoul 03722, Republic of Korea

E-mail: kimjb@yonsei.ac.kr

Prof. U-Syn Ha

Department of Urology, Seoul St. Mary’s Hospital, College of Medicine, The Catholic University of Korea,

222 Banpo-daero, Seocho-gu, Seoul 06591, Republic of Korea

E-mail: ushamd@catholic.ac.kr

**Table of contents:**

**Fig. S1.** Schematic of the manual pump designed to transport working fluid into the artificial bladder, facilitating the voiding process. The pump structure includes two check valves to ensure unidirectional flow and prevent backflow to the fluid reservoir. The pump’s configuration allows for a flow rate sufficient to expel all stored urine within 10 pumps. The flat base panel of the pump enhances pressure transmission, which is critical for intra-body insertion. The compact design prioritizes miniaturization, making the pump suitable for implantation alongside the artificial bladder.

**Fig. S2.** Fabrication and integration process of the RFID-based urine fullness detection system for the artificial bladder. (a) Construction of the capacitance-based sensor using a curved electrode and a pair of electrodes attached to the inner wall. The capacitor electrodes, made from conductive nickel fabric, are coated with a thin PDMS layer to minimize interference with the bladder’s septum membrane. This configuration allows the capacitance to vary as the bladder fills and the curved electrode deforms, thereby enabling urine volume detection. (b) Integration of the sensor components, including the antenna coil, RFID chip, and capacitor electrodes, onto the outer wall mold. The outer wall of the artificial bladder is then molded with these components, completing the sensor assembly and wiring. This system enables wireless monitoring of urine fullness without the need for an internal power source, as it operates by receiving power from an external RFID reader.

**Fig. S3.** Surgical procedure for the implantation of the artificial bladder in a pig model. (a) The pig's native bladder was removed through a transabdominal approach after dissection and securing of the bilateral ureters and urethra. (b) The artificial bladder was positioned in the orthotopic location on the pelvic floor, replacing the native bladder. (c) The ureters were sutured to the inlet of the artificial bladder, and the urethra was connected to the outlet annulus, where a Foley catheter was indwelled for urinary management. (d) After ensuring hemostasis and placing a drain tube on the pelvic floor, the abdominal incision was closed. Post-operative care included hydration, infection prevention, nutrition, wound care, and monitoring following standard laboratory animal care protocols. This study was conducted with approval from the Daegu Gyeongbuk Medical Innovation Foundation Care and Use Committee and adhered to the institutional guidelines for the humane treatment of laboratory animals

**Fig. S4.** Optimized design model and process of the voiding pump. The design objectives and objective functions include maximizing the fluid discharge volume during a single pressurization and miniaturizing the pump. Five design variables influencing the two objective functions were specified: pump thickness, internal diameter of the pump, internal height of the pump, socket length, and tube diameter. The constraint condition was set to achieve a fluid discharge volume sufficient to expel all stored urine within 10 pumps, and the optimal design was carried out accordingly

**Fig. S5.** Design and integration of the RFID-based urine fullness sensor.
(a) Schematic (left) and photographic image (right) of the passive LC resonant circuit composed of an RFID chip, variable capacitor, and antenna coil. The capacitor is formed by a curved nickel fabric electrode and fixed electrodes, whose capacitance changes in response to bladder deformation. (b) Physical placement of the sensing system within the artificial bladder. The sensor is embedded in the working fluid partition along the flat inner wall. The overlapping area of the capacitor electrodes is positioned approximately 2 cm from the top of the bladder. Electrical connections between the capacitor and RFID chip are established using silver paste to ensure secure signal transmission.

**Fig. S6.** Misalignment tolerance of the RFID-based bladder fullness sensor. (a) Experimental setup used to evaluate the effect of angular and distance misalignment between the implanted RFID bladder fullness sensor and the external patch antenna. (b) Measured signal amplitude (ADC peak value) at varying angular offsets (0°, 10°, 20°, and 30°) and distances (10–13 cm) between the antenna and sensor under identical excitation conditions. The corresponding relative power losses were –1.0 dB, –1.9 dB, and –3.5 dB for 10°, 20°, and 30° angular misalignments, and –1.6 dB, –2.9 dB, and –3.6 dB for distance increments of 11 cm, 12 cm, and 13 cm, respectively. The sensor was experimentally confirmed to operate reliably down to –3.6 dB, indicating robust wireless communication even under moderate misalignment conditions. These results confirm that the RFID-based sensing system maintains stable operation within realistic positional tolerances expected during practical use.

**Fig. S7.** Quantitative evaluation of the RFID-based bladder fullness sensor. The performance of the RFID-based bladder fullness sensor was assessed by measuring shifts in resonance frequency in response to increasing urine volume. The artificial bladder used in this experiment had a total capacity of 100 mL, and the sensor was designed to activate at approximately 75 mL. Across three independent trials, the sensor consistently activated within the 70–75 mL range, confirming the accuracy of the design threshold. It should be noted that during testing, additional wiring and measurement interfaces may have introduced parasitic capacitance, resulting in slightly lower measured resonance frequencies compared to fully implanted conditions. Nonetheless, the consistent activation range demonstrates the reliability and reproducibility of the sensor for detecting bladder fullness.

**Fig. S8.** Representative microscopic images from the ISO 10993-5 cytotoxicity evaluation of the Parylene-C coated artificial bladder component using L-929 fibroblasts. The blank control, test sample extract, and negative control groups exhibited normal cell morphology and high cell density without evidence of cytopathic effects, whereas the positive control group showed severe cell damage and reduced cell viability. According to the ISO 10993-5 qualitative morphological grading criteria, the test sample exhibited a cytotoxicity grade of 0, confirming its non-cytotoxicity and biocompatibility.

**Fig. S9.** Accelerated aging test of the artificial bladder under simulated implantation conditions (a) Bar graph demonstrating the input and output volumes of 60 mL of artificial urine from five independent experiments conducted before and accelerated aging and (a) Discrepancy between input and output volumes, calculated as |Output-Input|/Input x 100%. The artificial bladder underwent an accelerated aging test simulating one year of implantation, conducted at 80 °C and 100% relative humidity for 19 days. These conditions were chosen to replicate a moist physiological environment under harsh conditions while preventing thermal deformation. Stability was evaluated through five independent experiments using artificially colored yellow fluid (artificial urine). For each test, 60 mL of artificial urine was infused, and both infused and discharged volumes were measured before and after accelerated aging. All tests showed that the input and output volumes remained consistent around the 60 mL target, with discrepancies less than 1.5%, well within the ±5% acceptance criterion, indicating that the artificial bladder maintained normal function throughout the aging process.

**Fig. S10.** Comparison of urinary calcification between non-coated and Parylene-C coated artificial bladder groups. Absorbance at 550 nm was measured from fluid samples collected after *in vivo* implantation to assess mineral deposition. In the urine compartment, the Parylene-C coated group (red) exhibited significantly lower absorbance compared to the non-coated group (blue), indicating effective suppression of urinary calcification by the Parylene-C barrier. In the working fluid compartment, absorbance remained negligible in both groups, suggesting that no cross-contamination or leakage occurred between the urine and working fluid compartments. These findings confirm both the efficacy of the coating and the functional integrity of the septum membrane. Data are presented as mean ± standard deviation (n = 5). ******p < 0.01, unpaired two-tailed t-test.

**Fig. S11.** Clarification on open system during testing. During the urine storage phase (left), only the working fluid return valve is open, while all other valves remain closed, effectively sealing the urine storage chamber. In this state, pressure is balanced through its interaction with the open working fluid chamber. During the voiding phase (right), the working fluid valve is closed and only the urine outlet valve is open, reversing the configuration. The working fluid chamber becomes sealed, and pressure is applied to the urine chamber to initiate discharge. Throughout both phases, valve control ensures that the system functions as a closed-loop hydraulic circuit.

**Fig. S12.** The anatomical placement of the artificial bladder and working fluid reservoir for human implantation. The artificial bladder and the working fluid reservoir are positioned in separate anatomical compartments, divided by the abdominal muscle layer. Sagittal views at approximately 50% and 100% urine fill levels are shown. To enable complete voiding, the volume of the working fluid reservoir must match that of the artificial bladder.

**Fig. S13.** Implantation sites of the manual pump and on/off valve in the proposed artificial bladder system for human use. The manual pump and the on/off valve are designed to be implanted in the scrotal pouch, following current clinical practices used in inflatable penile prostheses. To prevent retrograde flow of urine toward the kidneys caused by back pressure from the reservoir, one-way check valves are positioned at the junctions between the ureters and the artificial bladder.

**Table S1.** Material properties comparison. PDMS is a well-established biocompatible material, but its higher stiffness (Shore A ~43) limits its suitability for repeated manual compression. Dragon Skin™ 30, with a lower Shore hardness (A ~30) and higher elasticity, was selected to ensure comfortable and durable manual actuation over time. Dragon Skin™ 30 also passed ISO 10993-5 cytotoxicity tests, indicating acceptable short-term biocompatibility.

**Movie S1.** Verification of the design and deformation behavior of the septum membrane. The septum membrane deforms with minimal resistance in response to fluid accumulation. Urine can be stored with only a few cm H₂O of hydrostatic pressure, demonstrating the membrane’s high compliance and low threshold deformation characteristics.

**Movie S2.** A demonstration of the working principle of the artificial bladder. The video illustrates the storage, voiding, and sensing functions. It shows the septum membrane's movement as urine fills the bladder, the displacement of the working fluid into the reservoir, and the activation of the external manual pump to expel stored urine. The sensing system is also depicted, highlighting how it detects urine fullness and triggers an external signal.

**Movie S3.** Verification of the RFID-based urine volume sensing system in the artificial bladder. This video demonstrates the functionality of the wireless RFID-based urine fullness sensor, which detects bladder filling by monitoring capacitance changes. When the volume of urine stored in the bladder reaches a critical point, the sensor transmits a signal to an external RFID reader. The RFID reader that receives the signal notifies the user through a red LED. The system was tested using pork belly tissue to simulate human conditions, confirming reliable operation and a stable wireless communication range of up to 10 cm.

**Movie S4.** *In vitro* simulation of the urine storage phase of the artificial bladder. The video shows how the septum membrane moves outward as the bladder fills with urine (yellow liquid), displacing the working fluid (blue liquid) into the reservoir. The bladder demonstrates excellent compliance and stable pressure during storage.

**Movie S5.** *In vitro* simulation of the urine voiding phase of the artificial bladder. The video illustrates the external pump's action, pushing the working fluid back into the bladder. This movement of the septum membrane enables the effective expulsion of stored urine through the urethra, demonstrating controlled and efficient voiding.

**Movie S6.** *In vivo* urodynamic evaluation of the artificial bladder at six weeks post-implantation in a mini pig model. The video shows the dynamic operation of the artificial bladder during storage and voiding. As urine fills the bladder, the working fluid is displaced into the reservoir, and when the bladder is full, the manual pump is activated, expelling urine efficiently. The video supports the data in Fig. 6, confirming good compliance, effective storage and voiding of urine.


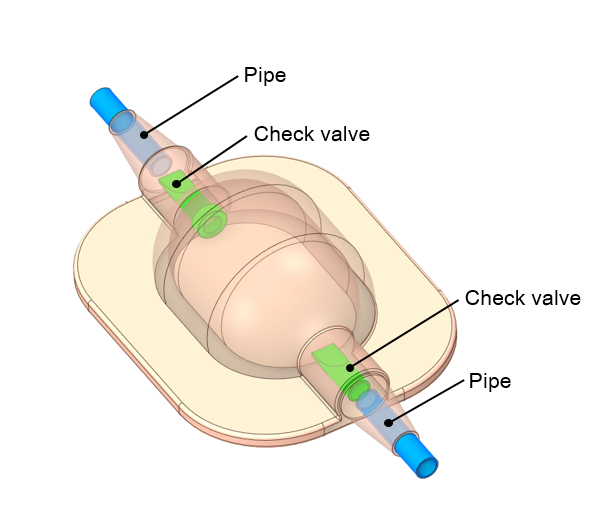


**Fig. S1.** Schematic of the manual pump designed to transport working fluid into the artificial bladder, facilitating the voiding process. The pump structure includes two check valves to ensure unidirectional flow and prevent backflow to the fluid reservoir. The pump’s configuration allows for a flow rate sufficient to expel all stored urine within 10 pumps. The flat base panel of the pump enhances pressure transmission, which is critical for intra-body insertion. The compact design prioritizes miniaturization, making the pump suitable for implantation alongside the artificial bladder.

**Fig. S2.** Fabrication and integration process of the RFID-based urine fullness detection system for the artificial bladder. (a) Construction of the capacitance-based sensor using a curved electrode and a pair of electrodes attached to the inner wall. The capacitor electrodes, made from conductive nickel fabric, are coated with a thin PDMS layer to minimize interference with the bladder’s septum membrane. This configuration allows the capacitance to vary as the bladder fills and the curved electrode deforms, thereby enabling urine volume detection. (b) Integration of the sensor components, including the antenna coil, RFID chip, and capacitor electrodes, onto the outer wall mold. The outer wall of the artificial bladder is then molded with these components, completing the sensor assembly and wiring. This system enables wireless monitoring of urine fullness without the need for an internal power source, as it operates by receiving power from an external RFID reader.


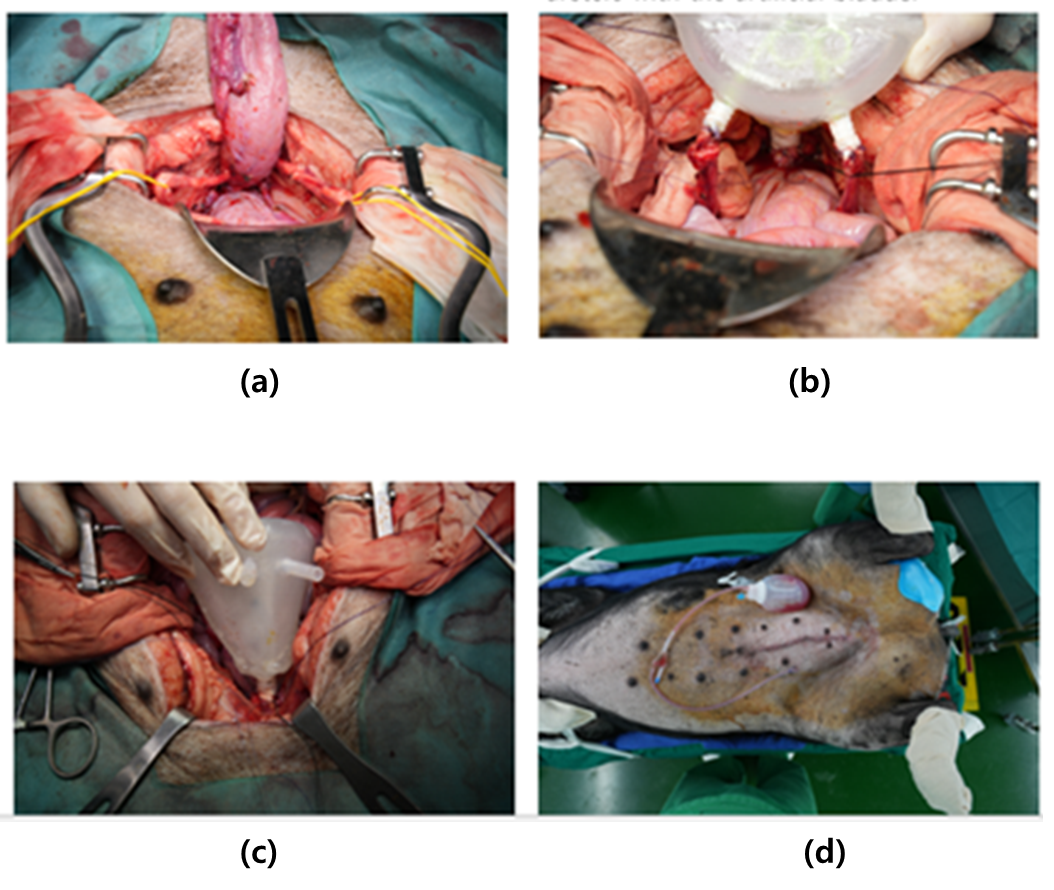


**Fig. S3.** Surgical procedure for the implantation of the artificial bladder in a pig model. (a) The pig's native bladder was removed through a transabdominal approach after dissection and securing of the bilateral ureters and urethra. (b) The artificial bladder was positioned in the orthotopic location on the pelvic floor, replacing the native bladder. (c) The ureters were sutured to the inlet of the artificial bladder, and the urethra was connected to the outlet annulus, where a Foley catheter was indwelled for urinary management. (d) After ensuring hemostasis and placing a drain tube on the pelvic floor, the abdominal incision was closed. Post-operative care included hydration, infection prevention, nutrition, wound care, and monitoring following standard laboratory animal care protocols. This study was conducted with approval from the Daegu Gyeongbuk Medical Innovation Foundation Care and Use Committee and adhered to the institutional guidelines for the humane treatment of laboratory animals

**
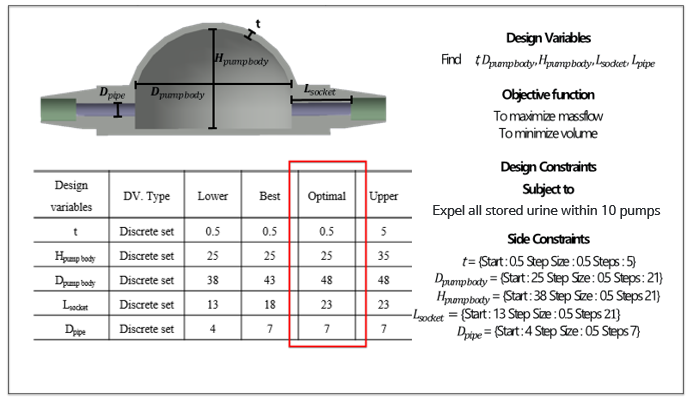
**

**Fig. S4.** Optimized design model and process of the voiding pump. The design objectives and objective functions include maximizing the fluid discharge volume during a single pressurization and miniaturizing the pump. Five design variables influencing the two objective functions were specified: pump thickness, internal diameter of the pump, internal height of the pump, socket length, and tube diameter. The constraint condition was set to achieve a fluid discharge volume sufficient to expel all stored urine within 10 pumpings, and the optimal design was carried out accordingly


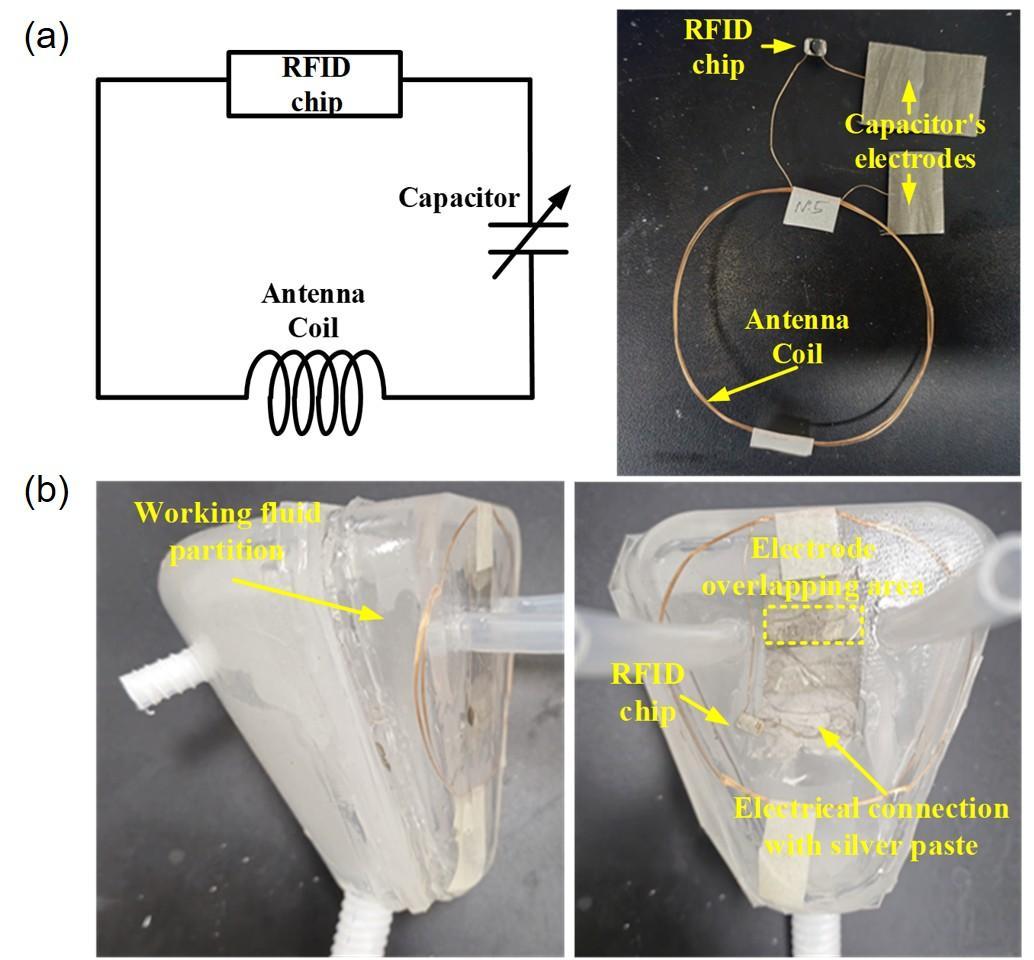


**Fig. S5.** Design and integration of the RFID-based urine fullness sensor.
(a) Schematic (left) and photographic image (right) of the passive LC resonant circuit composed of an RFID chip, variable capacitor, and antenna coil. The capacitor is formed by a curved nickel fabric electrode and fixed electrodes, whose capacitance changes in response to bladder deformation. (b) Physical placement of the sensing system within the artificial bladder. The sensor is embedded in the working fluid partition along the flat inner wall. The overlapping area of the capacitor electrodes is positioned approximately 2 cm from the top of the bladder. Electrical connections between the capacitor and RFID chip are established using silver paste to ensure secure signal transmission.

**
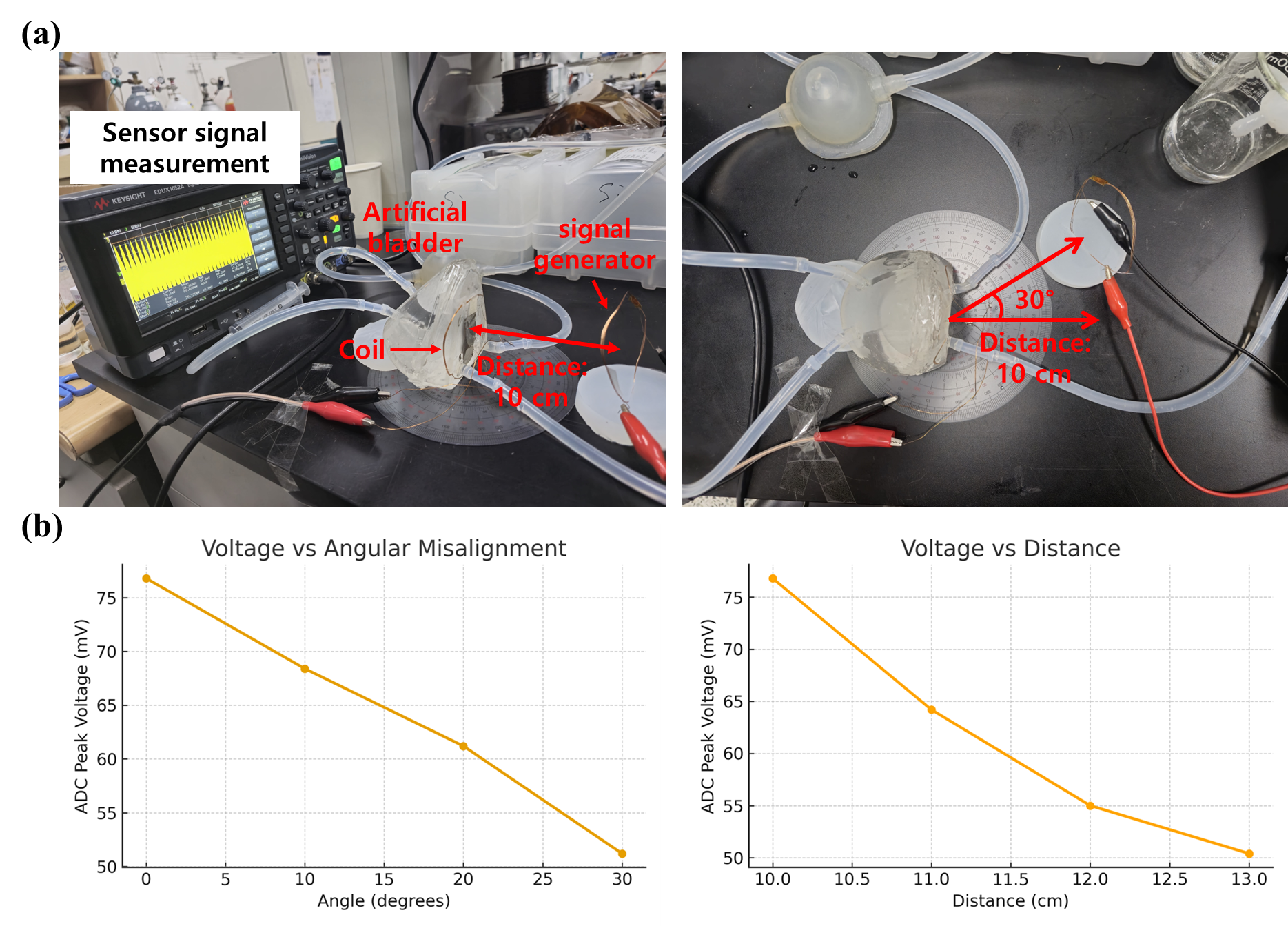
**

**Fig. S6.** Misalignment tolerance of the RFID-based bladder fullness sensor. (a) Experimental setup used to evaluate the effect of angular and distance misalignment between the implanted RFID bladder fullness sensor and the external patch antenna. (b) Measured signal amplitude (ADC peak value) at varying angular offsets (0°, 10°, 20°, and 30°) and distances (10–13 cm) between the antenna and sensor under identical excitation conditions. The corresponding relative power losses were –1.0 dB, –1.9 dB, and –3.5 dB for 10°, 20°, and 30° angular misalignments, and –1.6 dB, –2.9 dB, and –3.6 dB for distance increments of 11 cm, 12 cm, and 13 cm, respectively. The sensor was experimentally confirmed to operate reliably down to –3.6 dB, indicating robust wireless communication even under moderate misalignment conditions. These results confirm that the RFID-based sensing system maintains stable operation within realistic positional tolerances expected during practical use.

**
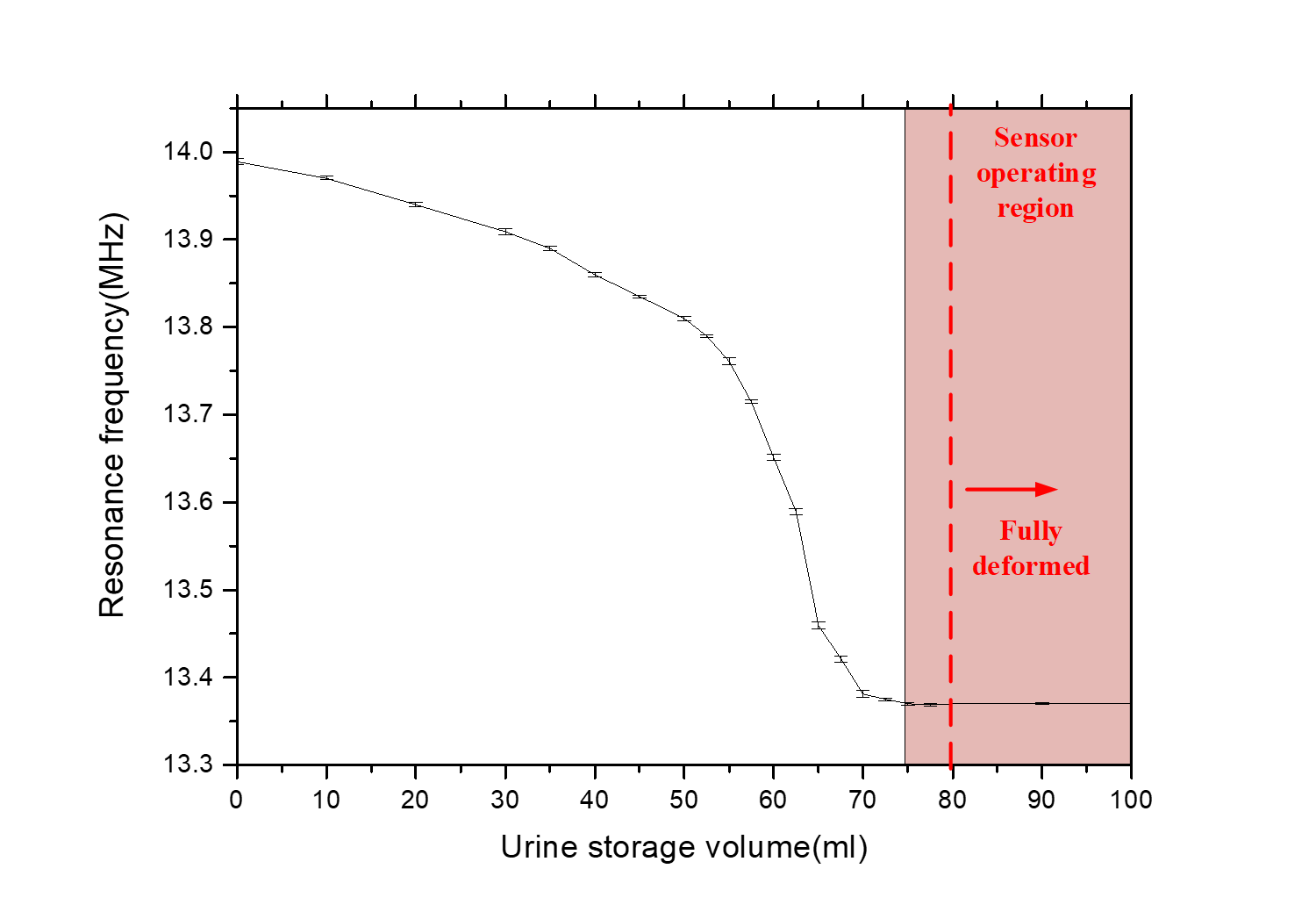
**

**Fig. S7.** Quantitative evaluation of the RFID-based bladder fullness sensor. The performance of the RFID-based bladder fullness sensor was assessed by measuring shifts in resonance frequency in response to increasing urine volume. The artificial bladder used in this experiment had a total capacity of 100 mL, and the sensor was designed to activate at approximately 75 mL. Across three independent trials, the sensor consistently activated within the 70–75 mL range, confirming the accuracy of the design threshold. It should be noted that during testing, additional wiring and measurement interfaces may have introduced parasitic capacitance, resulting in slightly lower measured resonance frequencies compared to fully implanted conditions. Nonetheless, the consistent activation range demonstrates the reliability and reproducibility of the sensor for detecting bladder fullness.


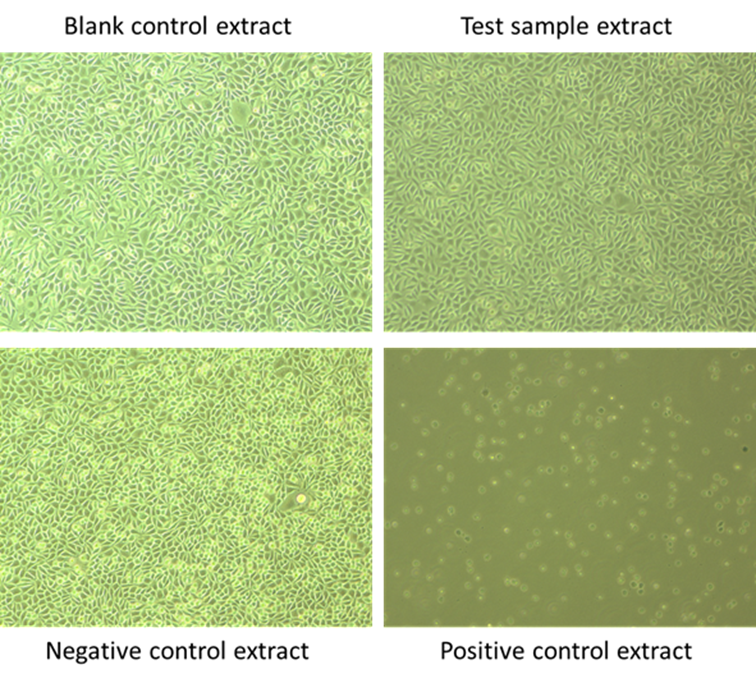


**Fig. S8.** Representative microscopic images from the ISO 10993-5 cytotoxicity evaluation of the Parylene-C coated artificial bladder component using L-929 fibroblasts. The blank control, test sample extract, and negative control groups exhibited normal cell morphology and high cell density without evidence of cytopathic effects, whereas the positive control group showed severe cell damage and reduced cell viability. According to the ISO 10993-5 qualitative morphological grading criteria, the test sample exhibited a cytotoxicity grade of 0, confirming its non-cytotoxicity and biocompatibility.

**
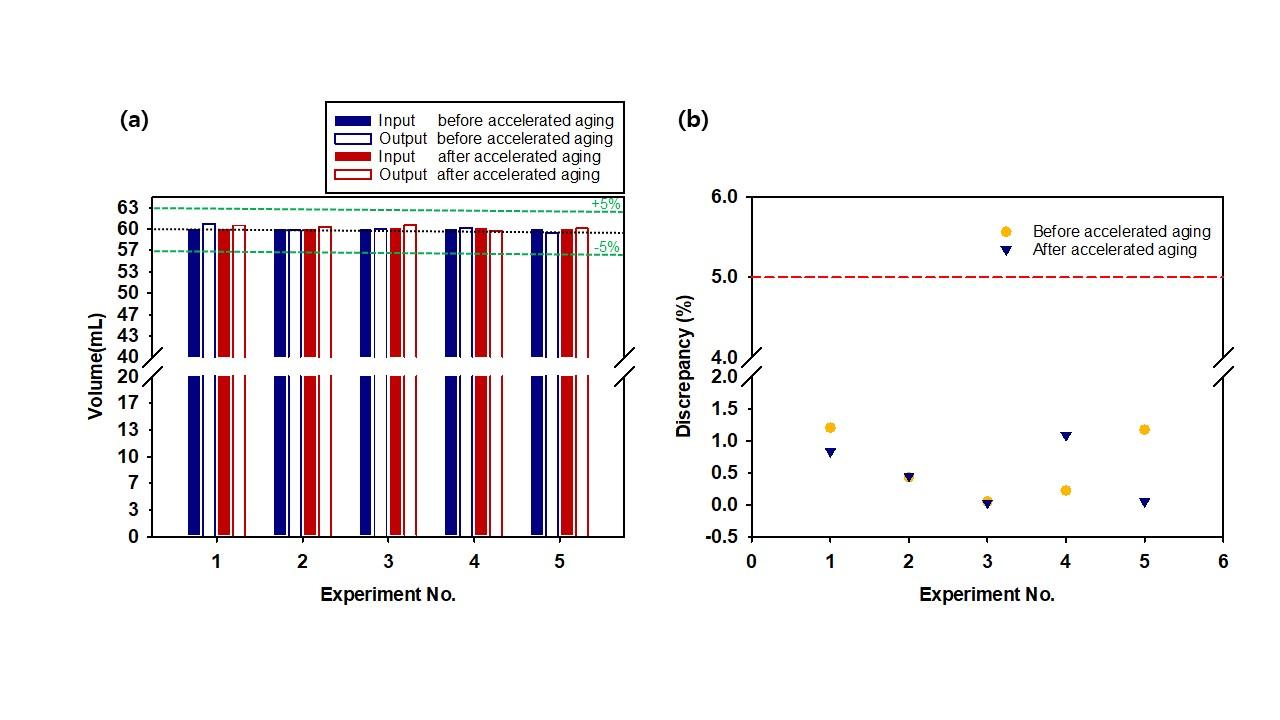
**

**Fig. S9.** Accelerated aging test of the artificial bladder under simulated implantation conditions (a) Bar graph demonstrating the input and output volumes of 60 mL of artificial urine from five independent experiments conducted before and accelerated aging and (a) Discrepancy between input and output volumes, calculated as |Output-Input|/Input x 100%. The artificial bladder underwent an accelerated aging test simulating one year of implantation, conducted at 80 °C and 100% relative humidity for 19 days. These conditions were chosen to replicate a moist physiological environment under harsh conditions while preventing thermal deformation. Stability was evaluated through five independent experiments using artificially colored yellow fluid (artificial urine). For each test, 60 mL of artificial urine was infused, and both infused and discharged volumes were measured before and after accelerated aging. All tests showed that the input and output volumes remained consistent around the 60 mL target, with discrepancies less than 1.5%, well within the ±5% acceptance criterion, indicating that the artificial bladder maintained normal function throughout the aging process.

**
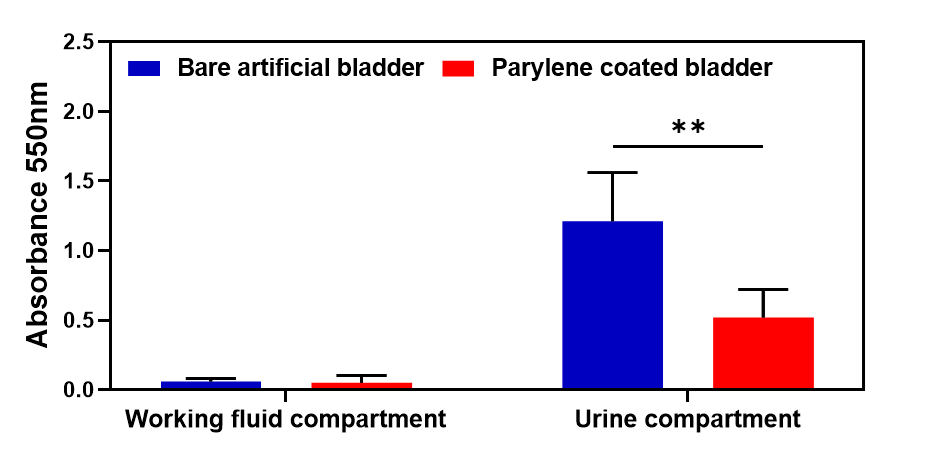
**

**Fig. S10.** Comparison of urinary calcification between non-coated and Parylene-C coated artificial bladder groups. Absorbance at 550 nm was measured from fluid samples collected after *in vivo* implantation to assess mineral deposition. In the urine compartment, the Parylene-C coated group (red) exhibited significantly lower absorbance compared to the non-coated group (blue), indicating effective suppression of urinary calcification by the Parylene-C barrier. In the working fluid compartment, absorbance remained negligible in both groups, suggesting that no cross-contamination or leakage occurred between the urine and working fluid compartments. These findings confirm both the efficacy of the coating and the functional integrity of the septum membrane. Data are presented as mean ± standard deviation (n = 5). ******p < 0.01, unpaired two-tailed t-test.

**
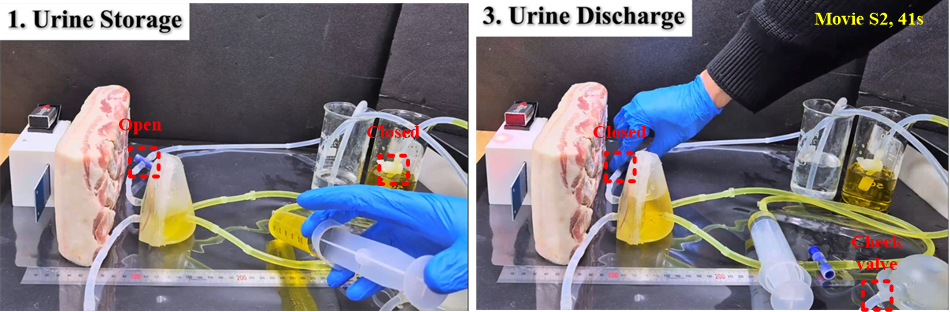
**

**Fig. S11.** Clarification on open system during testing. During the urine storage phase (left), only the working fluid return valve is open, while all other valves remain closed, effectively sealing the urine storage chamber. In this state, pressure is balanced through its interaction with the open working fluid chamber. During the voiding phase (right), the working fluid valve is closed and only the urine outlet valve is open, reversing the configuration. The working fluid chamber becomes sealed, and pressure is applied to the urine chamber to initiate discharge. Throughout both phases, valve control ensures that the system functions as a closed-loop hydraulic circuit.


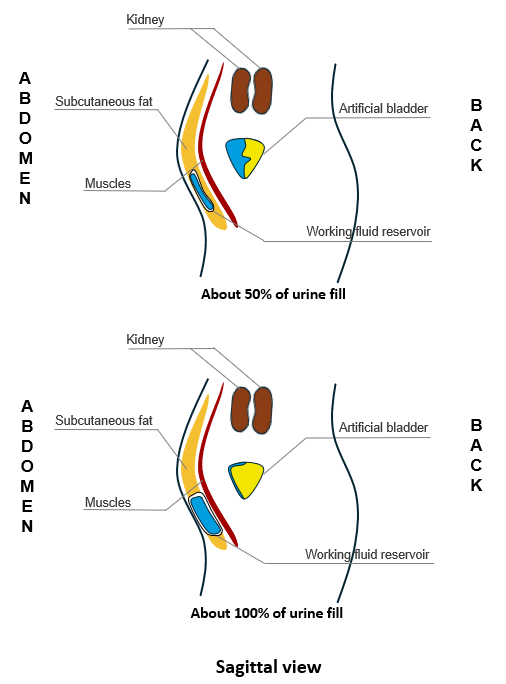


**Fig. S12.** The anatomical placement of the artificial bladder and working fluid reservoir for human implantation. The artificial bladder and the working fluid reservoir are positioned in separate anatomical compartments, divided by the abdominal muscle layer. Sagittal views at approximately 50% and 100% urine fill levels are shown. To enable complete voiding, the volume of the working fluid reservoir must match that of the artificial bladder.

**
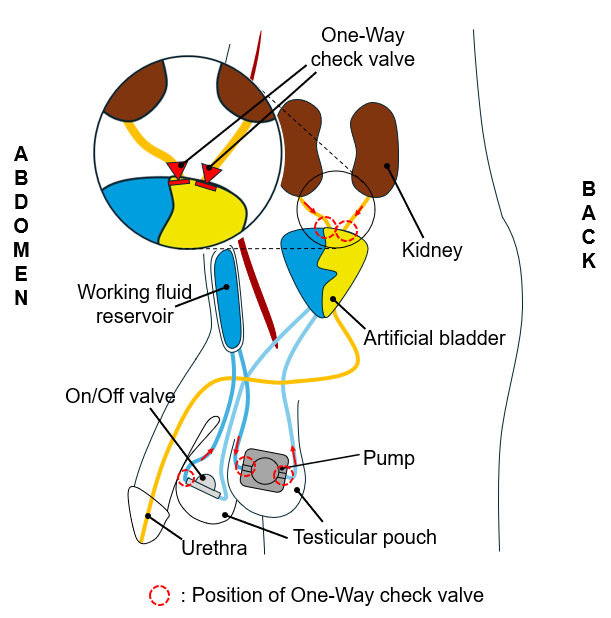
**

**Fig. S13.** The implant sites of the manual pump and the on/off valve for human implant. The manual pump and the on/off valve switch were designed to be implanted in the scrotal pouch, mimicking current clinical practice with artificial penile prostheses. To prevent reflux of urine to the kidneys due to possible back pressure from the reservoir, the One-Way check valve will be installed at the entrance from the kidney to the bladder.

| Physical properties | Sylgard 184 | Dragon Skin™ 30 |
| --- | --- | --- |
| Durometer Shore A | 43 A | 30 A |
| Tensile strength | 980 PSI (6.7 MPa) | 500 PSI (0.59 MPa) |
| Elongation at break | 122–296% | 364% |

**Table S1.** Material properties comparison. PDMS is a well-established biocompatible material, but its higher stiffness (Shore A ~43) limits its suitability for repeated manual compression. Dragon Skin™ 30, with a lower Shore hardness (A ~30) and higher elasticity, was selected to ensure comfortable and durable manual actuation over time. Dragon Skin™ 30 also passed ISO 10993-5 cytotoxicity tests, indicating acceptable short-term biocompatibility.
