# Supplementary material for: Preclinical development of engineered biomaterial-based artificial bladder demonstrating core functions in a large-animal orthotopic model
Source: Mater Today Bio. 2026 Jun 29;39:103404. doi: 10.1016/j.mtbio.2026.103404 (PMC13355429; doi:10.1016/j.mtbio.2026.103404)
Supplement: Multimedia component 2 [file mmc2.pdf]

TEST REPORT

REISSUE

Issue No.		TG-24-282	Receipt No.	G-24-233
Test Completion date		12/04/2024	Date of Receipt	07/04/2024
Product Name		-	Manufacturing report (declare) number	-
Client	Name	Kim Jin Ho	Company name	YeungNam University
	Address	(38541) Room 309, Mechanical Engineering Building, YeungNam University, 280, Daehak-ro, Gyeongsan-si, Gyeongsangbuk-do<br>(Tel: 010-8726-7781 Fax: Email: happykim96@yu.ac.kr)		
Manufacturer	Name	YeungNam University		
	Country	Republic of Korea		
	Address	(38541) Room 309, Mechanical Engineering Building, YeungNam University, 280, Daehak-ro, Gyeongsan-si, Gyeongsangbuk-do		
Purpose		For assignment verification		
Standard/Test method		According to the criteria provided by the client		
Test Result		See the test result report		
Affirmation		Tested by<br>Name : Deokkyu Yoon (signature)	Technical Manager<br>Name : Jeong-Mu Cheon (signature)	

This test report is only valid on the test subject materials provided by the client. This report shall not be reproduced in full or in part without the written approval of the Deagu-Gyeongbuk Medical Innovation Foundation Medical Device Development Center in advance. Also, this report is not related to KS Q ISO/IEC 17025 and KOLAS accreditation.

05 / 23 / 2025

Attachment : Test result report

Daegu-Gyeongbuk Medical Innovation Foundation

ADRESS : 80, Cheombok-ro, Dong-gu, Daegu, Republic of Korea
TEL : +82-53-790-5690, FAX : +82-53-790-5519

REISSUE

Test Items and Results

No	Requirement - Test	Results	Judgment
Stability Test			
1	**Stability test of artificial bladder**<br>The stability of the artificial bladder system is evaluated by assessing its durability and performance characteristics through accelerated aging under harsh conditions that simulate the physiological environment.	See attachment 1	Suitability

REISSUE

[Attachment 1]

Stability Test Report of Artificial Bladder

Item Name	Artificial bladder
Model	-
Lot No.	-
Date	12/04/2024

Test Start Date	11/14/2024
Test End Date	12/04/2024

	Tested by	Approved by
Position	Test personnel	Test manager
Name	Deokkyu Yoon	Jeong-Mu Cheon

Daegu-Gyeongbuk Medical Innovation Foundation

Medical Device Development Center

Test Results

1. Introduction

1.1 Purpose

The purpose of this test is to evaluate the stability of the artificial bladder system by assessing its durability and performance characteristics through accelerated aging under harsh conditions that simulate the physiological environment.

1.2 Test Period

Test initiated : 11/14/2024

Test completed : 12/04/2024

1.3 Test Standards

- The criteria provided by the client
- Reference standard : MFDS Notification No. 2023-34, Stability test standards for medical devices

2. Test Materials

2.1 Test Article

The test articles provided by the client.

Item name	Model name	Lot No.	Storage condition	Client
Artificial bladder	-	-	Room temperature	YeungNam University

2.2 Test material

The test materials provided by the client.

Material name	Composition
Artificial urine	Sterile saline dyed yellow
Working fluid	Distilled water dyed blue

2.3 Equipment

Name	No.	Date of Cal.	The due date of next Cal.
Electronic balance (Sartorius, ENTRIS 2202I-1S)	DGMIF-B191	07/22/2024	07/22/2025
Temperature & Humidity chamber (JEIO Tech, TH-G-180)	DGMIF-S045	09/05/2024	09/05/2025

3. Test Methods

3.1 Accelerated Aging Process

1) The artificial bladder, as an implantable organ, was subjected to accelerated aging under harsh conditions simulating the physiological environment to confirm its post-implantation stability.

2) Accelerated aging conditions

① Ambient Temperature(T_{RT}): The ambient temperature was set to 37 ℃, corresponding to normal human body temperature at the time of implantation.

② Accelerated Aging Temperature(T_{AA}): The accelerated aging temperature was set to 80 ℃, which is the maximum temperature that Parylene-C, the coating material for the artificial bladder and working fluid pump, can withstand without thermal deformation.

③ Reaction rate coeffecient(Q_{10}): 2

④ To simulate continuous exposure to bodily fluids, the artificial bladder and the working fluid pump were immersed in sterile saline solution and sealed in a glass bottle throughout the accelerated aging process. After the accelerated aging process was completed, the saline solution was removed and a performance test was conducted.

3) Accelerated Aging Periods

In this test, the real-time aging period was defined as one year (365 days), and the corresponding accelerated aging period was determined to be 19 days based on the following equation.

- A_{AF}(Accelerated Aging Factor) = $Q_{10}^{[(TAA-TRT)/10]}$ ≒ 19.70
- A_{AT}(Accelerated Aging Time) = 365 days/A_{AF} ≒ 19 days
- Accelerated Aging Periods : 11/14/2024 – 12/03/2024 (19 days)

3.2 Durability Inspection

1) Before and after accelerated aging, the appearance of the artificial bladder and the working fluid pump were visually inspected to identify any deformation or damage.

2) During the performance test of the artificial bladder and working fluid pump, both before and after accelerated aging, the artificial bladder and the working fluid pump were conducted to detect any leakage of artificial urine and working fluid, including at all connecting parts.

3.3 Performance Characteristic Test

1) To verify fluid flow and confirm the normal operation in the artificial bladder, the artificial urine (yellow) and the working fluid (blue) were prepared. A schematic diagram of the artificial bladder system is shown in Figure 1.

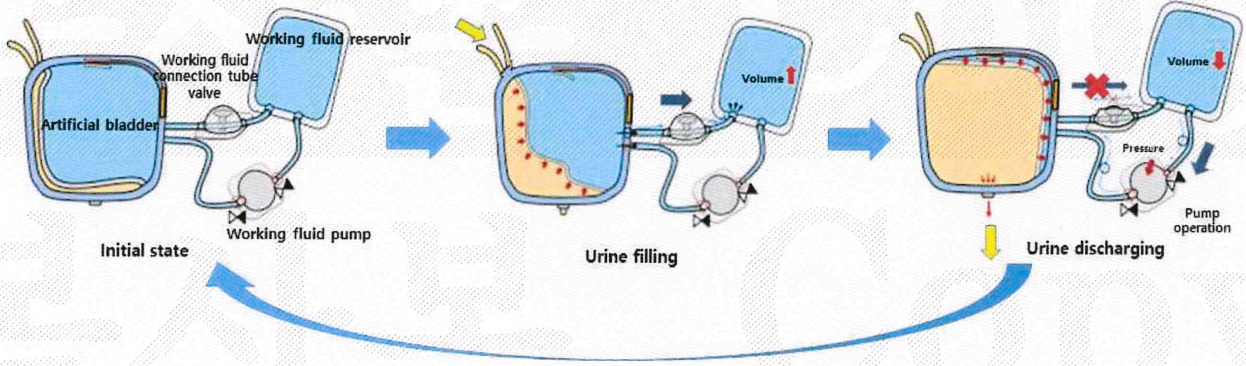

<Figure 1. A schematic diagram of the artificial bladder system>

2) The artificial bladder, the working fluid pump, and the working fluid reservoir were connected using a connecting tubes to enable circulation of the working fluid. The artificial bladder system connected for performance characteristics testing is shown in Figure 2.

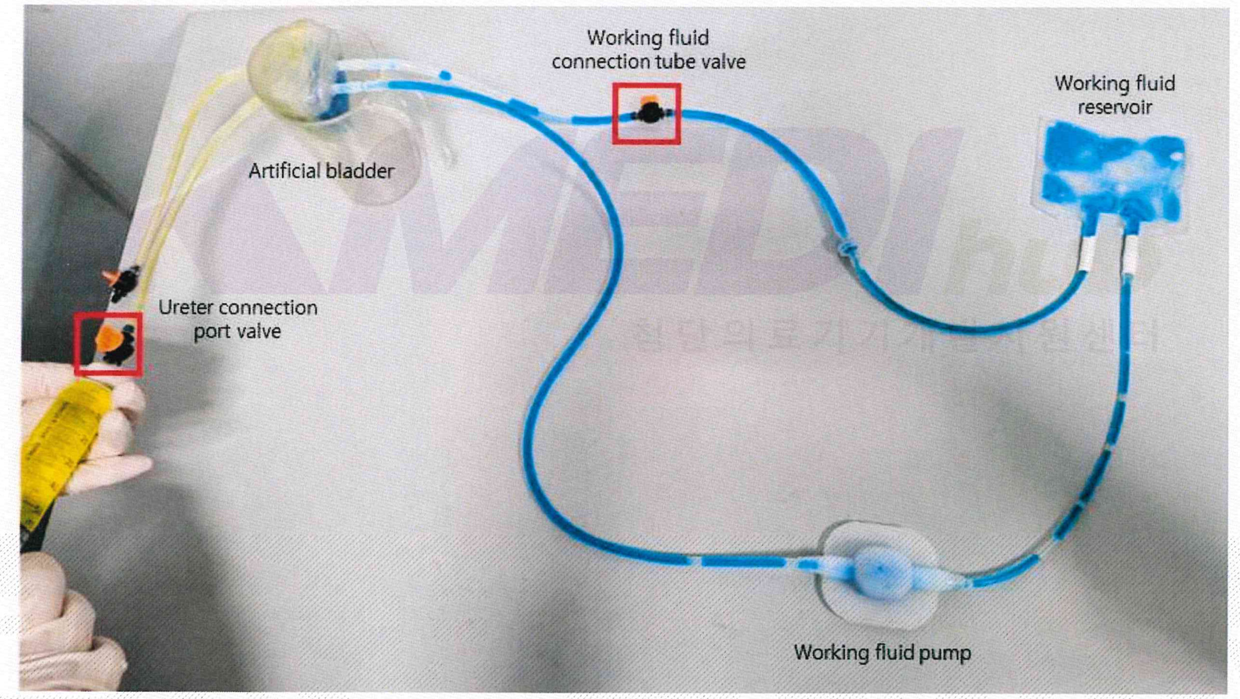

<Figure 2. Artificial bladder system configuration for performance testing>

3.3.1 Verification of the Normal Operation

1) The normal operation was verified by measuring urine injection and discharge volumes, both before and after accelerated aging.

2) The normal operation of the artificial bladder system was evaluated based on the discrepancy between injection and discharge volumes.

3.3.2 Measurement of Urine Injection and Discharge Volumes

1) The maximum volume capacity of the artificial bladder is approximately 70 mL. When the maximum volume of artificial urine is injected, the leakage occurs due to the pressure of urine, not the working fluid. Therefore, 60 mL of urine, representing 80 to 90 % of the maximum volume capacity, was injected and discharged during the test.
2) 60 g of artificial urine was filled into the syringe. (60 g ≒ 60 mL)
3) The syringe was connected to the ureteral connection port. After opening both ureteral connection port valve and the working fluid connection tube, artificial urine was slowly injected.
4) After completing the artificial urine injection, the valves on both the ureteral connection port and the working fluid connection tube were closed. Then, the working fluid pump was manually operated to allow working fluid to flow into the artificial bladder (Figure 3).
5) The working fluid was pressed until the working fluid is stopped flowing due to pressure (Figure 4).
6) The discharge volume of urine was measured using an electronic balance.
7) Steps 2) through 6) were repeated 5 times to measure the volumes of urine injected and discharged, and the discrepancies were calculated.

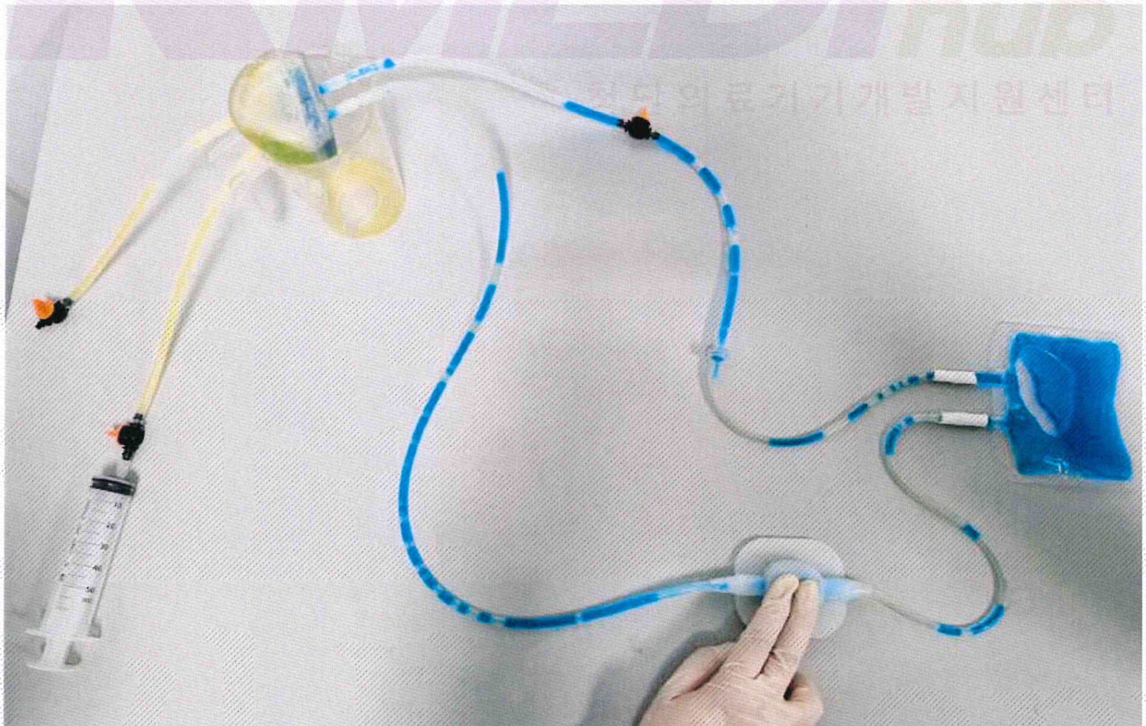

<Figure 3. Operation of the artificial bladder working fluid pump>

REISSUE

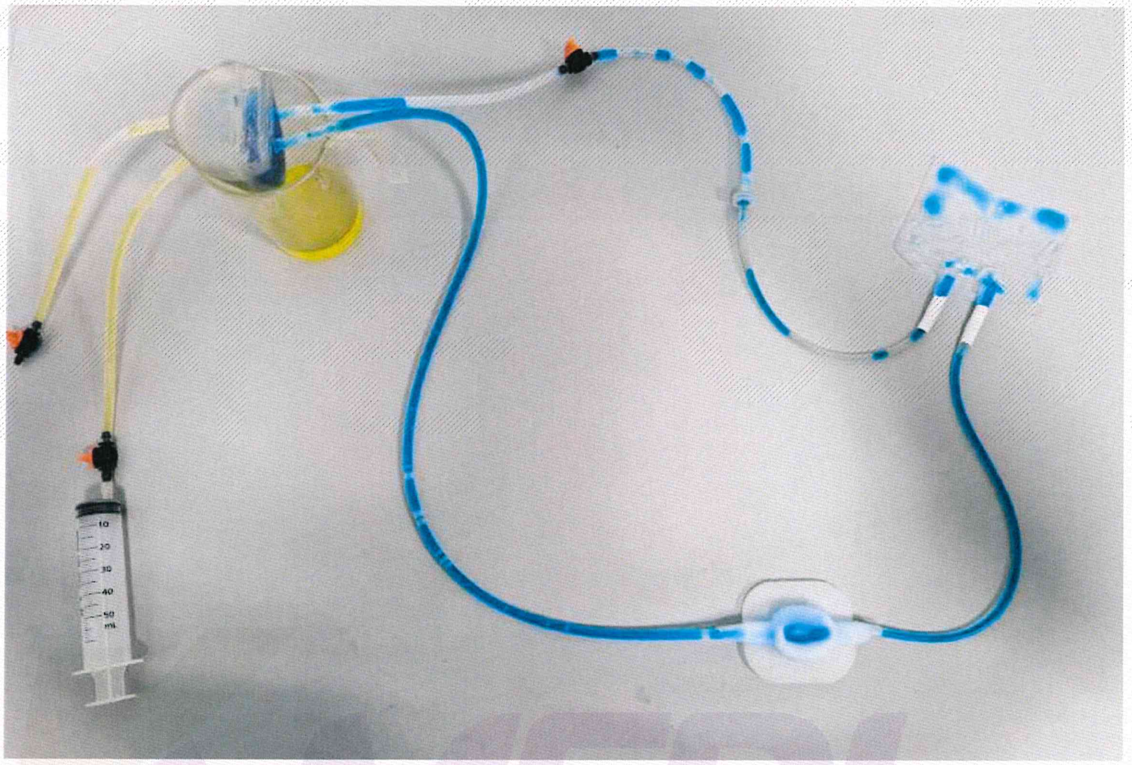

<Figure 4. Urine discharge according to the operation of the artificial bladder working fluid pump>

4. Evaluation Criteria

4.1 Durability Check

When visually inspecting the appearance of the artificial bladder and working fluid pump before and after accelerated aging, no deformation or damage should be observed. Additionally, during the performance characteristics test, there should be no leakage of the artificial urine or the working fluid, including at all connecting parts.

4.2 Verification of the Normal Operation, and Measurement of Urine Injection/Discharge Volumes.

When tested according to the prescribed test methods, the artificial bladder system should operate normally. The discrepancy between the injected and discharged urine volumes should be within 5%.

5. Test Results

5.1 Durability

Inspection item	Before accelerated aging	After accelerated aging
Deformation	No deformation	No deformation
Damage	No damage	No damage
Leakage	No leakage	No leakage
Judgment	Suitability	Suitability

5.2 Verification of the Normal Operation

No.	Before accelerated aging	After accelerated aging
# 1	Normal operation	Normal operation
# 2	Normal operation	Normal operation
# 3	Normal operation	Normal operation
# 4	Normal operation	Normal operation
# 5	Normal operation	Normal operation
Judgment	Suitability	Suitability

5.3 Measurement of Urine Injection/Discharge Volumes

Items	No.	Injection Volume (mL)	discharge Volume (mL)	Discrepancy (mL)	Discrepancy (%)	Judgment
Before accelerated aging	# 1	60.00	60.72	+ 0.72	1.20	Suitability
	# 2	59.99	59.73	− 0.26	0.43	
	# 3	60.00	59.97	− 0.03	0.05	
	# 4	60.00	60.13	+ 0.13	0.22	
	# 5	59.99	59.29	− 0.70	1.17	
After accelerated aging	# 1	59.98	60.48	+ 0.50	0.83	Suitability
	# 2	60.00	60.27	+ 0.27	0.45	
	# 3	60.01	60.59	− 0.02	0.03	
	# 4	60.01	59.64	− 0.65	1.08	
	# 5	60.00	60.03	+ 0.03	0.05	

6. Conclusion and Discussion

To evaluate the stability of the artificial bladder for an implantable organ, as requested by YeungNam University, the durability, the normal operation, and urine injection/discharge volume measurement before and after the accelerated aging were assessed under harsh conditions simulating one year of use.

As a results, the artificial bladder met all evaluation criteria for durability and performance, confirming its stability under the one year accelerated aging conditions.

In the future, it is expected that the long-term stability of the artificial bladder is expected to be further evaluated through extended period of accelerated aging tests.

7. Records

All raw data pertaining to this study and a copy of the final report are to be retained by and designated to K-MEDI hub archive files for a period of 5 years. END.

KMEDIhub 첨단의료기기개발지원센터

복사본 Copy
